# Short- and long-term mortality prediction after an acute ST-elevation myocardial infarction (STEMI) in Asians: A machine learning approach

**DOI:** 10.1371/journal.pone.0254894

**Published:** 2021-08-02

**Authors:** Firdaus Aziz, Sorayya Malek, Khairul Shafiq Ibrahim, Raja Ezman Raja Shariff, Wan Azman Wan Ahmad, Rosli Mohd Ali, Kien Ting Liu, Gunavathy Selvaraj, Sazzli Kasim

**Affiliations:** 1 Bioinformatics Division, Institute of Biological Sciences, Faculty of Science, University of Malaya, Kuala Lumpur, Malaysia; 2 Cardiology Department, Faculty of Medicine, Universiti Teknologi MARA (UiTM), Shah Alam, Malaysia; 3 Cardiac Vascular and Lung Research Institute, Universiti Teknologi MARA (UiTM), Shah Alam, Malaysia; 4 National Heart Association of Malaysia, Heart House, Kuala Lumpur, Malaysia; 5 Division of Cardiology, University Malaya Medical Centre, Kuala Lumpur, Malaysia; 6 Cardiac Vascular Sentral Kuala Lumpur, Kuala Lumpur, Malaysia; Kurume University School of Medicine, JAPAN

## Abstract

**Background:**

Conventional risk score for predicting short and long-term mortality following an ST-segment elevation myocardial infarction (STEMI) is often not population specific.

**Objective:**

Apply machine learning for the prediction and identification of factors associated with short and long-term mortality in Asian STEMI patients and compare with a conventional risk score.

**Methods:**

The National Cardiovascular Disease Database for Malaysia registry, of a multi-ethnic, heterogeneous Asian population was used for in-hospital (6299 patients), 30-days (3130 patients), and 1-year (2939 patients) model development. 50 variables were considered. Mortality prediction was analysed using feature selection methods with machine learning algorithms and compared to Thrombolysis in Myocardial Infarction (TIMI) score. Invasive management of varying degrees was selected as important variables that improved mortality prediction.

**Results:**

Model performance using a complete and reduced variable produced an area under the receiver operating characteristic curve (AUC) from 0.73 to 0.90. The best machine learning model for in-hospital, 30 days, and 1-year outperformed TIMI risk score (AUC = 0.88, 95% CI: 0.846–0.910; vs AUC = 0.81, 95% CI:0.772–0.845, AUC = 0.90, 95% CI: 0.870–0.935; vs AUC = 0.80, 95% CI: 0.746–0.838, AUC = 0.84, 95% CI: 0.798–0.872; vs AUC = 0.76, 95% CI: 0.715–0.802, p < 0.0001 for all). TIMI score underestimates patients’ risk of mortality. 90% of non-survival patients are classified as high risk (>50%) by machine learning algorithm compared to 10–30% non-survival patients by TIMI. Common predictors identified for short- and long-term mortality were age, heart rate, Killip class, fasting blood glucose, prior primary PCI or pharmaco-invasive therapy and diuretics. The final algorithm was converted into an online tool with a database for continuous data archiving for algorithm validation.

**Conclusions:**

In a multi-ethnic population, patients with STEMI were better classified using the machine learning method compared to TIMI scoring. Machine learning allows for the identification of distinct factors in individual Asian populations for better mortality prediction. Ongoing continuous testing and validation will allow for better risk stratification and potentially alter management and outcomes in the future.

## Introduction

Half of the global burden related to ischemic heart disease occurs within the Asia-Pacific region [1]. Prediction of mortality risks associated with the acute coronary syndrome (ACS) is often evaluated using risk scores such as the Thrombolysis in Myocardial Infarction (TIMI) or Global Registry of Acute Cardiac Events (GRACE) scores. These scores are extrapolated from studies with predominantly Caucasian patients with limited participation from Asia [2]. Asian countries tend to have younger patients with myocardial infarction, a higher burden of diabetes melitus, hypertension and renal failure as well as higher rates of delayed presentation for medical care [3, 4]. South-East Asia in particular is unique because of its heterogeneity due to inherent genetic variations in an already diverse group of multi-ethnic communities. Conventional risk scores may not be able to account for nuances related to the individual region in terms of disease burden, healthcare resources and available interventions.

The TIMI risk score is widely used due to its simplicity in calculation and accuracy in STEMI patients. TIMI scoring, unlike the GRACE score, was derived from patients with ST-segment elevation myocardial infarction (STEMI) only [5]. Studies using TIMI scores amongst Asians revealed a higher incidence of STEMI when compared to their Caucasian counterpart with somewhat similar mortality risk. This discrepancy is difficult to explain especially in the context of a higher disease burden amongst Asian patients.

Conventional cardiovascular disease (CVD) risk assessment models assume that risk factors have a linear relationship to clinical outcomes, leading to the oversimplification of a truly complex correlation. There is a need to develop models which consider these multiple risk factors and outcomes, including the use of machine learning (ML) algorithms [2, 6–8].

Current evidence supporting the use of ML over statistically-based models in mortality predictions include Logistic Regression (LR), Support Vector Machine (SVM) and Random Forest (RF). ML has been shown to outperform the conventional risk scoring model in population-specific mortality studies, post-STEMI, in countries like China, Israel and Korea [2, 7, 9].

To our knowledge, the development, and application of ML algorithms to predict short- and long-term mortality post-STEMI in a heterogeneous Asian population has yet to be reported. The study aims to identify factors and develop an ML model risk calculator that predicts short and long-term mortality in a heterogeneous South-East Asian population.

## Methods

### Study data

We used retrospective data from the Malaysian National Cardiovascular Database (NCVD-ACS) registry collected between 2006 until 2016. The NCVD registry was approved by the Medical Review & Ethics Committee (MREC), Ministry of Health (MOH) Malaysia in 2007 (Approval Code: NMRR-07-20-250). MREC waived informed patient consent for NCVD. The registry collects data on a standardised set of clinical, demographic, and procedural variables, along with outcomes, for consecutive patients treated at participating institutions [10, 11]. The study was also approved by the UITM ethic committee (Reference number: 600-TNCPI (5/1/6)) and the National Heart Association of Malaysia (NHAM) for data acquisition.

All patients from the ACS registry without exclusion were used including patients who received reperfusion (fibrinolysis, primary PCI (PPCI), angiography demonstrating spontaneous reperfusion, or urgent coronary artery bypass grafting (CABG)) for STEMI. In this context, STEMI was defined as persistent ST-segment elevation ≥ 1 mm in two contiguous electrocardiographic leads, or the presence of a new left bundle branch block in the setting of positive cardiac markers. 50 variables from a complete set of data were used in this study based on clinical recommendation. Categories of variables used were sociodemographic characteristics, CVD diagnosis and severity, CVD risk factors, CVD comorbidities, non-CVD comorbidities, biomarkers and medication used. The mortality time frame was calculated from first hospital admission for in-hospital, 30 days and 1-year. Confirmation of deaths was done yearly via record linkages with the Malaysian National Registration Department. The data collected by the registry does not include data on short term complication such as heart failure. The follow-up data points are meant to collect these variables but unfortunately are excessive in terms of missing values and hence we omitted this from the study. We focused our algorithm to policy changing endpoints for example hard endpoints such as death to increase the impact of the study. This is similarly done in other publications [2, 7, 9].

### Classification and sample pre-processing

We developed the ML using a complete set of data to ensure the validity of the findings. A total of 27,592 STEMI cases from the registry were collected 12,368 were identified as complete cases (with no missing values on predictors). Out of the 12 368 datasets, a total of 6299, 3130 and 2939 complete cases were used for in-hospital, 30-days and 1-year respectively for the model development. This rendered almost 50% complete cases of patients with a full predictor set of 50 variables for each time frame (9 continuous, 41 categorical) for the study (Table 1). Stratified random sampling of data was used [12]. Data were split for model development (70%) and validation (30%). We accessed the performance of ML and TIMI using a validation set that accounts for 30% of data for each time frame that is not used for model development.

**Table 1:** Patients characteristics for the in-hospital, 30-days and 1-year dataset.

Variables	Description	In-hospital				30 days				1-year			
		Total	Survival	Non-survival	*p*-value	Total	Survival	Non-survival	*p*-value	Total	Survival	Non-survival	*p*-value
N		6299	5961 (94.6)	338 (5.4)		3130	2878 (91.9)	252 (8.1)		2939	2516 (85.6)	423 (14.4)	
Age		55.8 ± 11.5	55.4 ± 11.3	63.8 ± 12.0	0.81	56.6 ± 11.7	56.0 ±11.4	64.2 ±12.5	0.054	56.6 ± 11.6	55.5 ± 11.2	62.8 ± 12.0	**0.028**
Race	Malay	3574 (56.7)	3365 (56.5)	209 (61.8)	**0.050**	1763 (56.3)	1608 (55.9)	155 (61.5)	**0.003**	1625 (55.3)	1370 (54.5)	255 (60.3)	**0.004**
	Chinese	1194 (19.0)	1126 (18.9)	68 (20.1)		552 (13.6)	498 (17.3)	54 (21.4)		531 (18.1)	453 (18.0)	78 (18.4)	
	Indian	1217 (19.3)	1170 (19.6)	47 (13.9)		640 (20.5)	602 (20.9)	38 (15.1)		610 (20.8)	530 (21.1)	80 (18.9)	
	Others	314 (5.0)	300 (5.0)	14 (4.1)		175 (5.6)	170 (5.9)	5 (2.0)		173 (5.9)	163 (6.5)	10 (2.4)	
Gender	Male	5417 (86.0)	5152 (86.4)	265 (78.4)	**<0.0001**	2681 (85.7)	2486 (86.4)	195 (77.4)	**<0.0001**	2533 (86.2)	2214 (88.0)	319 (75.4)	**<0.0001**
	Female	882 (14.0)	809 (13.6)	73 (21.6)		448 (14.4)	392 (13.6)	57 (22.6)		406 (13.8)	302 (12.0)	104 (24.6)	
Smoking status	Never	2003 (31.8)	1866 (31.3)	137 (40.5)	**<0.0001**	1053 (33.6)	941 (32.7)	112 (44.4)	**<0.0001**	977 (33.2)	786 (31.2)	191 (45.2)	**<0.0001**
	Former (quit tobacco > 30days)	1019 (16.2)	952 (16.0)	67 (19.8)		472 (15.1)	424 (14.7)	48 (19.0)		440 (15.0)	371 (14.7)	69 (16.3)	
	Current (tobacco < 30days)	3277 (52.0)	3143 (52.7)	134 (39.6)		1605 (51.3)	1513 (52.6)	92 (36.5)		1522 (51.8)	1359 (54.0)	163 (38.5)	
History of hypertension		3344 (53.1)	3112 (52.2)	232 (68.6)	**<0.0001**	1697 (54.2)	1538 (53.4)	159 (63.1)	**0.003**	1587 (54.0)	1316 (52.3)	271 (64.1)	**<0.0001**
History of diabetes		2482 (39.4)	2291 (38.4)	191 (56.5)	**<0.0001**	1271 (40.6)	1129 (39.2)	142 (56.3)	**<0.0001**	1187 (40.4)	945 (37.6)	242 (57.2)	**<0.0001**
Family history of premature cardiovascular disease		892 (14.2)	869 (14.6)	23 (6.8)	**<0.0001**	435 (13.9)	419 (14.6)	16 (6.3)	**<0.0001**	410 (14.0)	372 (14.8)	38 (9.0)	**0.0001**
History of myocardial infarction		625 (9.9)	580 (9.7)	45 (13.3)	**0.032**	299 (9.6)	271 (9.4)	28 (11.1)	0.380	278 (9.5)	231 (9.2)	47 (11.1)	0.210
Documented CAD		583 (9.3)	552 (9.3)	31 (9.2)	0.956	358 (11.4)	323 (11.2)	35 (13.9)	0.202	341 (11.6)	273 (10.9)	68 (16.1)	**0.002**
History of heart failure		124 (2.0)	109 (1.8)	15 (4.4)	**0.001**	56 (1.8)	49 (1.7)	7 (2.8)	0.217	49 (1.7)	32 (1.3)	17 (4.0)	**<0.0001**
Chronic lung disease		114 (1.8)	101 (1.7)	13 (3.8)	**0.004**	61 (1.9)	54 (1.9)	7 (2.8)	0.321	60 (2.0)	44 (1.7)	16 (3.8)	**0.006**
Chronic renal disease		191 (3.0)	158 (2.7)	33 (9.8)	**<0.0001**	104 (3.3)	77 (2.7)	27 (10.7)	**<0.0001**	98 (3.3)	52 (2.1)	46 (10.9)	**<0.0001**
Cerebrovascular disease		171 (2.7)	156 (2.6)	15 (3.3)	**0.045**	88 (2.8)	80 (2.8)	8 (3.2)	0.716	84 (2.9)	63 (2.5)	21 (5.0)	**0.005**
Heart rate		82.4 ± 21.1	81.7 ± 20.6	93.9 ± 26.6	**<0.0001**	82.9 ± 20.9	81.9 ± 20.0	94.5 ±27.0	**<0.0001**	82.6 ±20.6	81.1 ± 19.6	91.7 ± 24.2	**<0.0001**
Systolic blood pressure		132.8 ± 27.8	135.6 ± 27.4	120.4 ± 30.2	**0.011**	134.9 ± 28.2	135.6 ±28.0	126.4 ± 29.4	**<0.0001**	153.1 ± 28.0	135.9 ± 27.3	130.1 ± 3.1	**0.010**
Diastolic blood pressure		82.8 ± 94.1	83.4 ± 96.5	73.6 ± 20.2	0.965	81.3 ± 18.5	81.8 ±18.3	76.2 ±19.8	**<0.0001**	81.5 ± 18.4	82.1 ±18.1	78.4 ±20.0	0.066
Killip class	I	4300 (68.3)	4210 (70.6)	90 (26.6)	**<0.0001**	2072 (66.2)	1998 (69.4)	74 (29.4)	**<0.0001**	1980 (67.4)	1809 (71.9)	141 (40.4)	**<0.0001**
	II	1190 (18.9)	1132 (19.0)	58 (17.2)		558 (17.8)	506 (17.6)	52 (20.6)		512 (17.4)	413 (16.4)	99 (23.4)	
	III	237 (3.8)	200 (3.4)	37 (10.9)		128 (4.1)	98 (3.4)	30 (11.9)		110 (3.7)	71 (2.8)	39 (9.2)	
	IV	572 (9.1)	419 (7.0)	153 (45.3)		372 (11.9)	276 (9.6)	96 (38.1)		337 (11.5)	223 (8.9)	114 (27.0)	
Total cholesterol		5.4 ± 1.6	5.4 ± 1.6	4.8 ± 1.7	0.10	5.2 ± 1.4	5.3 ± 1.3	4.9 ± 1.6	**<0.0001**	5.2 ± 1.4	5.3 ± 1.3	4.9 ± 1.6	**0.005**
HDL		1.1±1.2	1.1 ± 1.2	1.0 ±0.3	0.952	± 0.4	±0.4	1.1 ± 0.3	0.140	1.1 ± 0.3	1.1 ±0.3	1.1 ±0.4	0.130
LDL		3.8 ± 10.7	3.8 ± 11.0	3.0 ± 1.4	0.706	3.5 ± 5.4	3.5 ± 5.6	3.1 ± 1.4	0.295	3.1 ± 1.2	3.4 ± 1.2	3.1 ±1.4	**0.026**
Triglycerides		1.8 ± 1.7	1.8 ± 1.7	1.7 ± 1.0	0.529	1.7 ± 0.9	1.7 ± 0.9	1.7 ±0.9	0.762	1.7 ± 0.9	1.7 ±0.9	1.6 ±0.8	0.206
Fasting blood glucose		8.7 ± 4.4	8.5 ± 4.1	12.3 ± 6.7	**<0.0001**	8.8 ± 4.4	8.6 ± 4.1	11.7 ±6.5	**<0.0001**	8.8 ± 4.2	8.4 ± 3.8	10.8 ±5.8	**<0.0001**
ECG abnormalities type	ST segment elevation ≥1mm in ≥2 contiguous limb leads	2804 (44.5)	2565 (44.6)	148 (43.8)	0.782	1518 (48.5)	1403 (48.7)	115 (45.6)	0.343	1437 (48.9)	1241 (49.3)	196 (46.3)	0.255
	ST segment elevation ≥2mm in ≥2 contiguous frontal leads or chest leads	3373 (59.9)	3563 (59.8)	210 (62.1)	0.389	1828 (58.4)	1664 (57.8)	164 (65.1)	**0.025**	1710 (58.2)	1447 (57.5)	263 (62.2)	0.072
	ST segment depression ≥0.5mm in ≥2 contiguous leads	627 (10.0)	589 (9.9)	38 (11.2)	0.416	280 (8.9)	254 (8.8)	26 (10.3)	0.426	267 (9.1)	219 (8.7)	48 (11.3)	0.080
	T-wave inversion ≥1mm	394 (6.3)	378 (6.3)	16 (4.7)	0.235	197 (6.3)	184 (6.4)	13 (5.2)	0.439	189 (6.4)	152 (6.0)	37 (8.7)	**0.036**
	Bundle branch block	138 (2.2)	111 (1.9)	27 (8.0)	**<0.0001**	72 (2.4)	56 (1.9)	18 (7.1)	**<0.0001**	59 (2.0)	38 (1.5)	21 (5.0)	**<0.0001**
ECG abnormalities location	Inferior leads: II, III, aVF	2998 (47.6)	2859 (48.0)	139 (41.1)	**0.014**	1520 (48.6)	1415 (49.2)	105 (41.7)	**0.022**	1433 (48.8)	1251 (49.7)	182 (43.0)	**0.011**
	Anterior leads: V1 to V4	3435 (54.5)	3233 (54.2)	202 (59.8)	**0.047**	1655 (52.9)	1498 (52.1)	157 (62.3)	**0.022**	1545 (52.6)	1287 (51.2)	258 (61.0)	**<0.0001**
	Lateral leads: I, aVL, V5 to V6	1396 (22.2)	1295 (21.7)	101 (29.9)	**<0.0001**	744 (23.8)	659 (22.9)	85 (33.7)	**<0.0001**	705 (24.0)	567 (22.5)	138 (32.6)	**<0.0001**
	True posterior: V1, V2	515 (8.2)	484 (8.1)	321 (9.2)	0.492	258 (8.2)	235 (8.2)	23 (9.1)	0.595	243 (8.3)	213 (8.5)	30 (7.1)	0.343
	Right ventricle: ST elevation in lead V4R	524 (8.3)	494 (8.3)	30 (8.9)	0.703	286 (9.1)	262 (9.1)	24 (9.5)	0.824	269 (9.2)	231 (9.2)	38 (9.0)	0.896
FB status		4530 (71.9)	4288 (71.9)	242 (71.6)	0.893	2144 (68.5)	1973 (68.6)	171 (67.9)	0.819	1989 (67.7)	1717 (68.2)	272 (64.3)	0.109
Cardiac catheterization		2950 (46.8)	2812 (47.2)	138 (40.8)	**0.045**	1727 (55.2)	1619 (56.3)	108 (42.9)	**<0.0001**	1629 (55.4)	1455 (57.8)	174 (41.1)	**<0.0001**
PCI		2414 (38.3)	2298 (38.6)	116 (34.3)	0.120	1396 (44.6)	1304 (45.3)	92 (36.5)	**0.007**	1323 (45.0)	1188 (47.2)	135 (31.9)	**<0.0001**
CABG		33 (0.5)	30 (0.5)	3 (0.9)	0.341	33 (1.1)	27 (0.9)	6 (2.4)	**0.032**	22 (0.7)	18 (0.7)	4 (0.9)	0.611
ASA		6180 (98.1)	5862 (98.3)	318 (94.1)	**<0.0001**	3070 (98.1)	2829 (98.3)	241 (95.6)	**0.003**	2883 (98.1)	2473 (98.3)	410 (96.9)	0.058
GP receptor inhibitor		173 (2.7)	162 (2.7)	11 (3.3)	0.557	62 (2.0)	56 (1.9)	6 (2.4)	0.635	58 (2.0)	51 (2.0)	7 (1.7)	0.611
Heparin		962 (15.3)	900 (15.1)	62 (18.3)	0.107	549 (17.5)	501 (17.4)	48 (19.0)	0.512	523 (17.9)	459 (18.2)	64 (15.1)	0.121
LMWH		1546 (24.5)	1450 (24.3)	96 (28.4)	**0.09**	479 (15.3)	414 (14.4)	65 (25.8)	**<0.0001**	406 (13.8)	313 (12.4)	93 (22.0)	**<0.0001**
Beta blockers		4066 (64.5)	3978 (66.7)	88 (26.0)	**<0.0001**	1896 (60.6)	1800 (62.5)	96 (38.1)	**<0.0001**	1754 (59.7)	1558 (61.9)	196 (46.3)	**<0.0001**
ACE inhibitor		3320 (52.7)	3251 (54.5)	69 (20.4)	**<0.0001**	1509 (48.2)	1452 (50.5)	57 (22.6)	**<0.0001**	1388 (47.2)	1268 (50.4)	120 (28.4)	**<0.0001**
Angiotensin II receptor blocker		181 (2.9)	176 (3.0)	5 (1.5)	0.115	61 (1.9)	55 (1.9)	6 (2.4)	0.605	52 (1.8)	43 (1.7)	9 (2.1)	0.564
Statin		6013 (95.5)	5713 (95.8)	300 (88.8)	**<0.0001**	3003 (95.9)	2774 (96.4)	229 (90.9)	**<0.0001**	2820 (96.0)	2433 (96.7)	387 (91.5)	**<0.0001**
Other lipid lowering agent		127 (2.0)	124 (2.1)	3 (0.9)	0.129	51 (1.6)	48 (1.7)	3 (1.2)	0.566	47 (1.6)	38 (1.5)	9 (2.1)	0.349
Diuretics		1349 (21.4)	1201 (20.1)	148 (43.8)	**<0.0001**	720 (23.0)	610 (21.2)	110 (43.7)	**<0.0001**	651 (22.2)	473 (18.8)	178 (42.1)	**<0.0001**
Calcium antagonist		367 (5.8)	352 (5.9)	15 (4.4)	0.263	183 (5.8)	176 (6.1)	7 (2.8)	**0.030**	161 (5.5)	139 (5.5)	22 (5.2)	0.787
Oral hypoglycaemic agent		1345 (21.4)	1312 (22.0)	33 (9.8)	**<0.0001**	597 (19.1)	567 (19.7)	30 (11.9)	**0.003**	546 (18.6)	478 (19.0)	68 (16.1)	0.153
Insulin		1658 (26.3)	1516 (25.4)	142 (42.0)	**<0.0001**	869 (27.8)	757 (26.3)	112 (44.4)	**<0.0001**	804 (27.3)	624 (24.8)	180 (42.6)	**<0.0001**
Anti-arrhythmic agent		313 (5.0)	276 (4.6)	37 (0.9)	**<0.0001**	178 (5.7)	144 (5.0)	34 (13.5)	**<0.0001**	151 (5.1)	114 (4.5)	37 (8.7)	**<0.0001**

Abbreviations: CAD = coronary artery disease, HDL = high-density lipoprotein, LDL = low-density lipoprotein, ECG = electrocardiogram, FB = fibrinolytic therapy, PCI = percutaneous coronary intervention, CABG = coronary artery bypass graft, ASA = acetylsalicylic acid (aspirin), GP = glycoprotein, LMWH = low-molecular-weight heparin, ACE = Angiotensin-converting enzyme.

Data are shown as n (%) for categorical variables and mean ± SD for continuous variables.

p value is statistically highly significant as p < 0.001.

### Model development and calibration

Prediction models post-STEMI were developed using three selected ML algorithms. Next, feature selection (see below) was carried out on the ranked variables in an ascending order iteratively [13]. 10-fold cross-validation was used to avoid overfitting for model development on the training set [14]. The prediction models were trained and tested for each iteration, and the models with the highest performance consisting of the least number of variables were selected. Predictive performances of the models were calculated using the validation dataset.

Secondary analyses were carried out after adding 15224 missing cases imputed using multivariable imputation using chained equations and predictive mean matching that yields a total of 27 592 cases [15]. This method imputes missing values based on real values from other cases where predicted values are closest. Our reference for incomplete dataset refers to missing sets of variables up to 50%. The missing dataset mentioned refers to patient characteristics and not outcome data. As our dataset is a prospective dataset, with retrospective data management, the level of missingness in values across all variables was completely random and beyond our control. The probability of missingness in our dataset depends neither on the observed values in any variable of the dataset nor on the unobserved part of the dataset.

Hence the dataset is classified as missing completely at random (MCAR) which is the highest level of randomness and it implies that the pattern of missing value is random and does not depend on any variable which may or may not be included in the analysis. We had complete data for all our outcomes. The models were tested with a similar validation dataset for ML models trained with a complete cases dataset.

### Machine learning algorithms and calibration

Supervised classification ML algorithms RF [16], SVM [17] and LR [18] were selected in this study. They are the classifiers that have resulted in high predictive performance compared to conventional methods in mortality studies [7, 19]. RF and SVM are black-box models (models without interpretability) meanwhile LR is a white-box model (model with good interpretability) [16, 18, 20]. The ML algorithms’ parameters were set to the optimized value to obtain higher predictive performance (S1 Table). Tuned hyperparameters improve ML model performance over the default setting provided [21]. The area under the receiver operating curve (AUC) was used as a predictive performance metric [22]. Additional performance metrics were accuracy, sensitivity, specificity, positive predictive value (PPV) and negative predictive value (NPV) for model calibration [23]. Paired resampled t-test was used to compare ML models predictive performances [12, 24].

### Feature selection

Feature selection is the process of ranking variables using classifier specific variable evaluator. RF, SVM and LR variable importance method were used to rank variables importance associated with outcome (survival / non-survival at in-hospital, 30 days, and 1-year).

Feature reduction involves the elimination of existing variables to a minimal set, which reduces training time, produces better results, and increases the accuracy of results. Next, sequential backward elimination (SBE) [13] was used in this study for feature reduction on the ranked variables in ascending order of importance iteratively from the ML variables importance methods. The ML prediction model is retrained and tested each time a variable is eliminated. The variable that causes a significant decrease in the AUC of the prediction model upon elimination based on the ranked variable list using the feature selection method is deemed as important. We selected the important variables and ranked them again and the elimination process is repeated until a model with a reduced number of variables and the highest AUC value is achieved.

RFE combined with ML classifiers have been used in various clinical dataset successfully [25–28]. We also used Recursive feature elimination (RFE) to find a minimal and best set of variables by removing the least important features and compare them with feature selection by ML methods (RF, SVM and LR) [29, 30].

### Comparison with TIMI score

Calculated TIMI scores were used from the NCVD registry for the validation data performance. TIMI score performance (AUC) was compared with the developed ML-based models using the validation set. We derived a graph to compare performance between ML and TIMI score based on cutoff points applicable in clinical practice and literature [31]. A high risk of death was defined as a probability risk of death of more than 8% similar to reported in Correia et al. [31].

Net reclassification improvement (NRI) was used to determine the changes in discrimination between the TIMI risk score for STEMI and ML algorithm. The NRI uses reclassification tables to examine whether there is an additive benefit gained from reclassifying patients using a different approach in mortality assessment. By calculating the NRI, we were able to quantify the degree to which the different mortality risk assessment approaches driving correct movement between categories. An NRI can be interpreted as the percentage by which the net classification has improved by using a new different approach. The NRI was used to evaluate the improvement in classification obtained by comparing the TIMI risk score for STEMI with ML for STEMI [32].

### Additional statistics

The results are expressed as mean and SD for continuous variable and as frequencies for categorical variables. Correlation analysis was carried out to identify a significant relationship between variables. Univariate analysis was performed using a Chi-Square test to identify significant variables and a two-sided independent student t-test (p < 0.05). The ML performance was compared using a pair-wise corrected resampled *t*-test [33, 34]. Statistical significance was considered if the p-value was less than 0.05.

### Software

R package (Version 3.5.2) was used in ML algorithm development. Statistical analysis was conducted using Statistical Package for Social Sciences (SPSS) program version 16.0 [35].

## Results

### Patient characteristics

A total of 27,592 STEMI patients were identified. Incomplete data made up 55.2% of patients enrolled. Table 1 illustrates patients’ characteristic used in this study on the complete dataset. The mean age was 56.6 (SD = 11.7). The majority of patients (~87%) were males. The overall mortality reported for in-hospital, 30 days and 1-year was 5.4%, 8.1% and 14.4%. There was a significant difference between survivors to non-survivors for in-hospital, 30-days and 1-year mortality in terms of gender, smoking status, diabetes, renal disease, heart rate, Killip class, fasting blood glucose, ECG abnormalities, beta-blocker, ACE inhibitor, statin, diuretics, insulin and anti-arrhythmic agent use (p < 0.0001 for all). S2 Table illustrates patient’s characteristics for secondary analysis using an imputed dataset. Both statistical analyses on the complete and imputed dataset are almost similar.

### ML prediction

Maximal predictive performances on the validation dataset were observed for ML models constructed using reduced and complete sets of variables compared to TIMI risk score using untouched 30% validation dataset (Table 2). TIMI only outperformed the RFE-LR model for 30 days and 1-year mortality. The best-selected ML model (SVMvarImp–SBE–SVM) performed better against TIMI based on the AUC value using the untouched 30% validation dataset (p < 0.0001 for all models). Detailed performance evaluation of the best ML model against TIMI risk score is presented in Table 3.

**Table 2:** The AUC of TIMI risk score and ML models with and without feature selection based on a 30% validation dataset.

Classifiers	The area under the ROC Curve (95% CI)		
	In-hospital	30 days	1-year
RF	0.86 (0.820–0.88)	0.83 (0.786–0.879)	0.78 (0.741–0.827)
RFvarImp-SBE-RF	0.87 (0.832–0.907)	0.85 (0.10–0.890)	0.80 (0.750–0.834)
RFE-RF	0.86 (0.821–0.893)	0.82 (0.772–0.872)	0.79 (0.748–0.833)
SVM	0.86 (0.824–0.895)	0.87 (0.831–0.912)	0.84 (0.801–0.877)
SVMvarImp-SBE-SVM	0.88 (0.846–0.910)	0.90 (0.870–0.935)	0.84 (0.798–0.872)
RFE-SVM	0.85 (0.811–0.887)	0.88 (0.837–0.920)	0.84 (0.806–0.880)
LR	0.88 (0.846–0.911)	0.85 (0.803–0.897)	0.76 (0.710–0.807)
LRstepwise—SBE-LR	0.89 (0.861–0.920)	0.85 (0.812–0.906)	0.80 (0.767–0.848)
RFE- LR	0.87 (0.842–0.897)	0.83 (0.783–0.882)	0.78 (0.737–0.826)
TIMI	0.81 (0.772–0.802)	0.80 (0.746–0.838)	0.76 (0.715–0.802)

Abbreviations:

RF = Random Forest.

RFvarImp-SBE-RF = RF variable importance with sequential backward elimination and RF classifier.

RFE-RF = Recursive feature elimination with RFclassifier.

SVM = Support Vector Machine.

SVMvarImp-SBE-SVM = SVM variable importance with sequential backward elimination and SVM classifier.

RFE-SVM = Recursive feature elimination with SVM classifier.

LR = Logistic Regression.

LRstepwise—SBE-LR = LR stepwise feature elimination and LR classifier.

RFE- LR = Recursive feature elimination with LR classifier.

TIMI = Thrombolysis in Myocardial Infarction.

**Table 3:** Additional performance metrics based on a 30% validation dataset for TIMI risk score and ML models with and without feature selection.

	PPV	NPV	Sensitivity	Specificity	Accuracy (Cl 95%)	Mcnemar’s test (p-value)
**In-hospital**						
Classifier						
RF	0.380	0.963	0.347	0.968	0.935 (0.923,0.946)	<0.0001
RFvarImp-SBE-RF	0.447	0.963	0.337	0.977	0.942 (0.931,0.952)	<0.0001
RFE-RF	0.350	0.963	0.347	0.964	0.931 (0.918,0.942)	<0.0001
SVM	0.242	0.976	0.614	0.892	0.877 (0.861, 0.891)	<0.0001
SVMvarImp-SBE-SVM	0.219	0.980	0.693	0.861	0.852 (0.835,0.868)	<0.0001
RFE-SVM	0.202	0.982	0.723	0.838	0.832 (0.815, 0.849)	<0.0001
LR	0.211	0.981	0.713	0.850	0.842 (0.825,0.858)	<0.0001
LRstepwise—SBE-LR	0.211	0.984	0.752	0.841	0.836 (0.814,0.852)	<0.0001
RFE- LR	0.185	0.978	0.663	0.834	0.825 (0.807,0.842)	<0.0001
TIMI	0.180	0.976	0.644	0.834	0.824 (0.806, 0.841)	<0.0001
**30 days**						
Classifier						
RF	0.389	0.946	0.373	0.949	0.903 (0.882,0.921)	<0.0001
RFvarImp-SBE-RF	0.341	0.948	0.413	0.930	0.889 (0.867,0.909)	<0.0001
RFE-RF	0.414	0.947	0.387	0.952	0.907 (0.887,0.925)	<0.0001
SVM	0.258	0.972	0.733	0.817	0.810 (0.784,0.835)	<0.0001
SVMvarImp-SBE-SVM	0.258	0.983	0.840	0.790	0.794 (0.767,0.820)	<0.0001
RFE-SVM	0.261	0.980	0.813	0.800	0.801 (0.774,0.829)	<0.0001
LR	0.248	0.973	0.747	0.803	0.799 (0.771,0.824)	<0.0001
LRstepwise—SBE-LR	0.281	0.974	0.747	0.834	0.827 (0.802,0.851)	<0.0001
RFE- LR	0.248	0.971	0.720	0.810	0.803 (0.776,0.828)	<0.0001
TIMI	0.245	0.962	0.627	0.832	0.816 (0.789, 0.840)	<0.0001
**1-year**						
Classifier						
RF	0.410	0.909	0.373	0.949	0.827 (0.801,0.852)	<0.0001
RFvarImp-SBE-RF	0.436	0.909	0.460	0.901	0.838 (0.811,0.861)	<0.0001
RFE-RF	0.425	0.913	0.492	0.889	0.8318 (0.805,0.856)	<0.0001
SVM	0.382	0.950	0.746	0.798	0.7909 (0.763,0.817)	<0.0001
SVMvarImp-SBE-SVM	0.357	0.950	0.754	0.773	0.771 (0.741,0.798)	<0.0001
RFE-SVM	0.387	0.953	0.762	0.798	0.793 (0.765,0.820)	<0.0001
LR	0.329	0.924	0.611	0.792	0.766 (0.737,0.794)	<0.0001
LRstepwise—SBE-LR	0.372	0.935	0.659	0.814	0.792 (0.764,0.818)	<0.0001
RFE- LR	0.344	0.926	0.619	0.802	0.776 (0.747,0.803)	<0.0001
TIMI	0.332	0.907	0.484	0.837	0.786 (0.758, 0.813)	<0.0001

**Fig 1:**
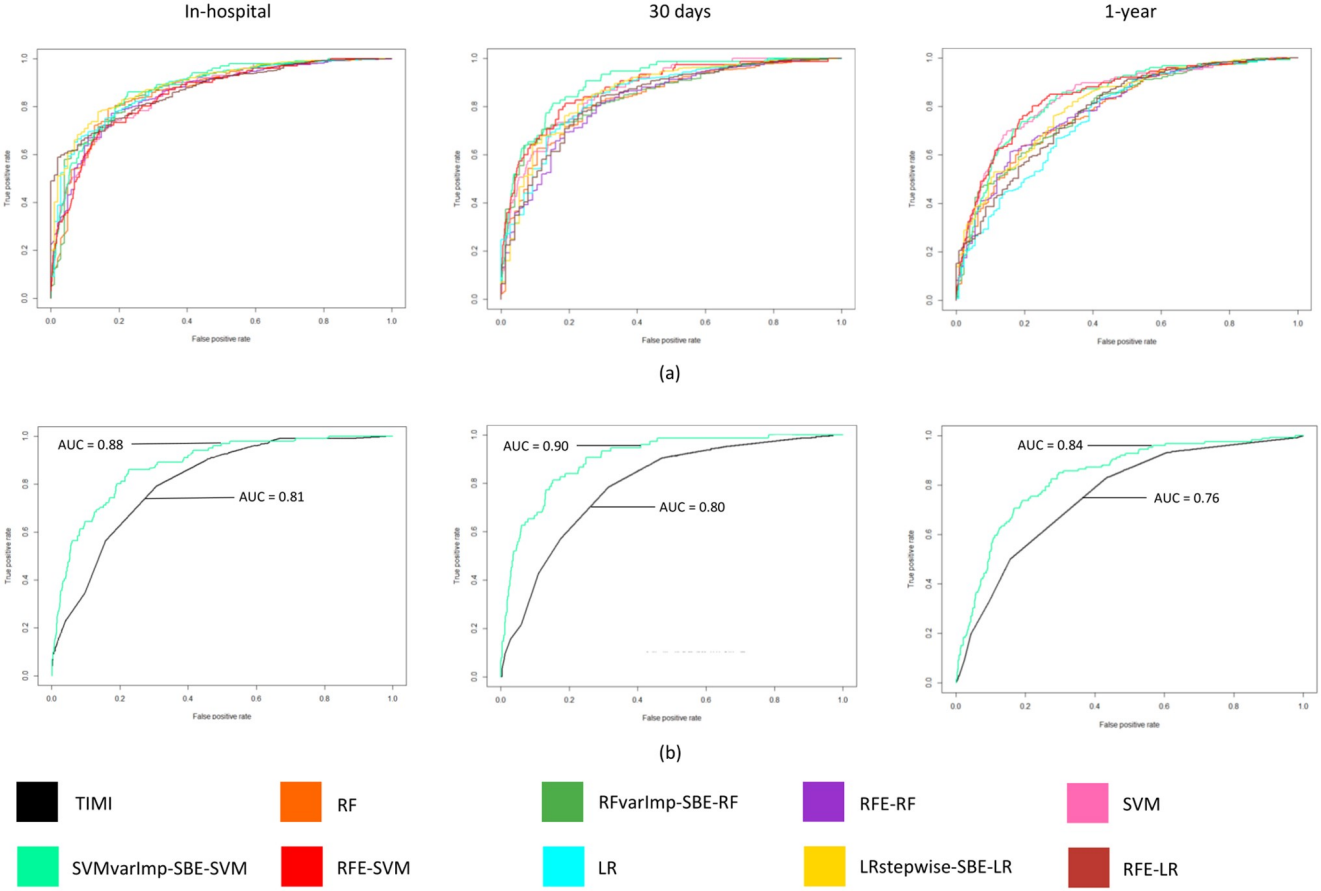
The receiver operating characteristics (ROC) curves of ML models and TIMI score based on a 30% validation dataset. ROC curves show the performance for In-hospital, 30 days and 1-year ML mortality prediction models (a). The ROC values for TIMI against the best ML model (SVMvarImp-SBS-SVM). Abbreviations: varImp = variable importance.

Fig 1(a) illustrates ML model performances and Fig 1(b) the best selected ML model against TIMI based on the AUC value using the untouched 30% validation dataset. Combination of SVMvarImp-SBE-SVM algorithm demonstrated the highest predictive performance with the least number of predictors for in-hospital, 30 days and 1-year model. There was no significant difference for in-hospital model for LRstepwise—SBE-LR (AUC = 0.89, 95% CI:0.861–0.920) with 24 predictors and SVMvarImp-SBE-SVM (AUC = 0.88, 95% CI: 0.846–0.910) with 15 predictors (p = 0.143; 95% CI, -0.026 to 0.004). 30-days ML mortality prediction for SVMvarImp–SBE–SVM (AUC = 0.90, 95% CI: 0.867–0.935) model reported no significant difference to RFE-SVM (AUC = 0.88,95% CI:0.837–0.920) (p = 0.115; 95% CI, -0.013 to 0.001). Model performances were observed to be similar (AUC = 0.84) and significantly better (p <0.0001) between the following 1-year mortality models (SVMvarImp vs SVM; 95% CI, 0.035 to 0.052, RFE-SVM vs SVM, 95% CI, 0.005 to 0.011, SVMvarImp-SBE-SVM vs RFE-SVM; 95% CI, 0.027 to 0.044). However, SVMvarImp-SBE-SVM model consisted of the least number of predictors (14 predictors) compared to SVM without feature selection (50 predictors) and RFE-SVM (44 predictors). Similar performance (p = 0.828; 95% CI, -0.007 to 0.009) were reported for LR with a reduced set of predictors (AUC = 0.85, 95% CI: 0.812–0.907) and a complete set of predictors for 30 days (AUC = 0.85, 95% CI: 0.803–0.897).

Secondary analysis on best ML models (SVMvarImp–SBE–SVM) performance trained with imputed data reported for in-hospital (AUC = 0.87, 95% CI: 0.845–0.912), 30day (AUC = 0.90, 95% CI: 0.857–0.923), and 1-year (AUC = 0.83, 95% CI: 0.796–0.871). Complete case ML model dataset resulted in an almost similar AUC result for in-hospital (AUC = 0.88, 95% CI: 0.846–0.910), 30 days (AUC = 0.90, 95% CI: 0.870–0.935), and 1-year mortality (AUC = 0.84, 95% CI: 0.798–0.872). For in-hospital and 30 days, the imputed and the complete case model is significant (p<0.0001; 95% CI, 0.011 to 0.018, p = 0.001; 95% CI, 0.004 to 0.016 respectively). As for 1-year model, it is not statistically significant between imputed and complete case model (p = 0.931; 95% CI, -0.006 to 0.005).

### Feature selection

RFE and SBE feature selection methods were combined with ML algorithms to construct predictive models with optimal performance (refer to methods). Initial ranking using all 50 variables for best model (SVMvarimp-SBE-SVM) using SVM variable importance is shown in S1–S3 Figs. SBE was then used to identify features that result in model optimal performances.

Common predictors observed for in-hospital, 30 days and 1-year mortality across all ML models in this study are (age, heart rate, Killip class, and fasting blood glucose). Diuretics were an additional common predictor for the best model (SVMvarImp-SBE-SVM). Age, heart rate and Killip class are identified as common predictors for the best ML model (SVMvarImp-SBE-SVM) in-hospital, 30 days and 1-year against TIMI (Table 4).

**Table 4:** Selected variables that resulted in optimum AUC for the best ML models (SVMvarImp-SBE-SVM) in in-hospital, 30-days, and 1-year against TIMI risk score variables.

Variables	Machine learning best model			TIMI Score
	In-hospital	30days	1-year	
Age	•	•	•	•
Race		•		
Smoking status			•	
Hypertension			•	•
Diabetes			•	•
Family history of premature CVD	•	•		
Chronic renal disease	•			
Heart rate	•	•	•	•
Systolic bp	•		•	•
Diastolic bp	•			
Killip class	•	•	•	•
HDL		•		
Fasting blood glucose	•	•	•	
Weight				•
ECG-type bundle branch block	•			•
ECG- location lateral lead	•			
Time to treatment				•
Cardiac catheterization	•	•		
PCI		•	•	
ASA		•		
Beta blockers	•	•		
ACE inhibitor			•	
Statin	•			
Diuretics	•	•	•	
Oral hypoglycaemic agent	•	•		
Insulin			•	

The best ML model (SVMvarImp–SBE–SVM) was converted into a short- and long-term online mortality calculator available at http://myheartstemi.uitm.edu.my/home.php.

### Comparison of ML to TIMI risk score when applied to validation dataset

**Fig 2:**
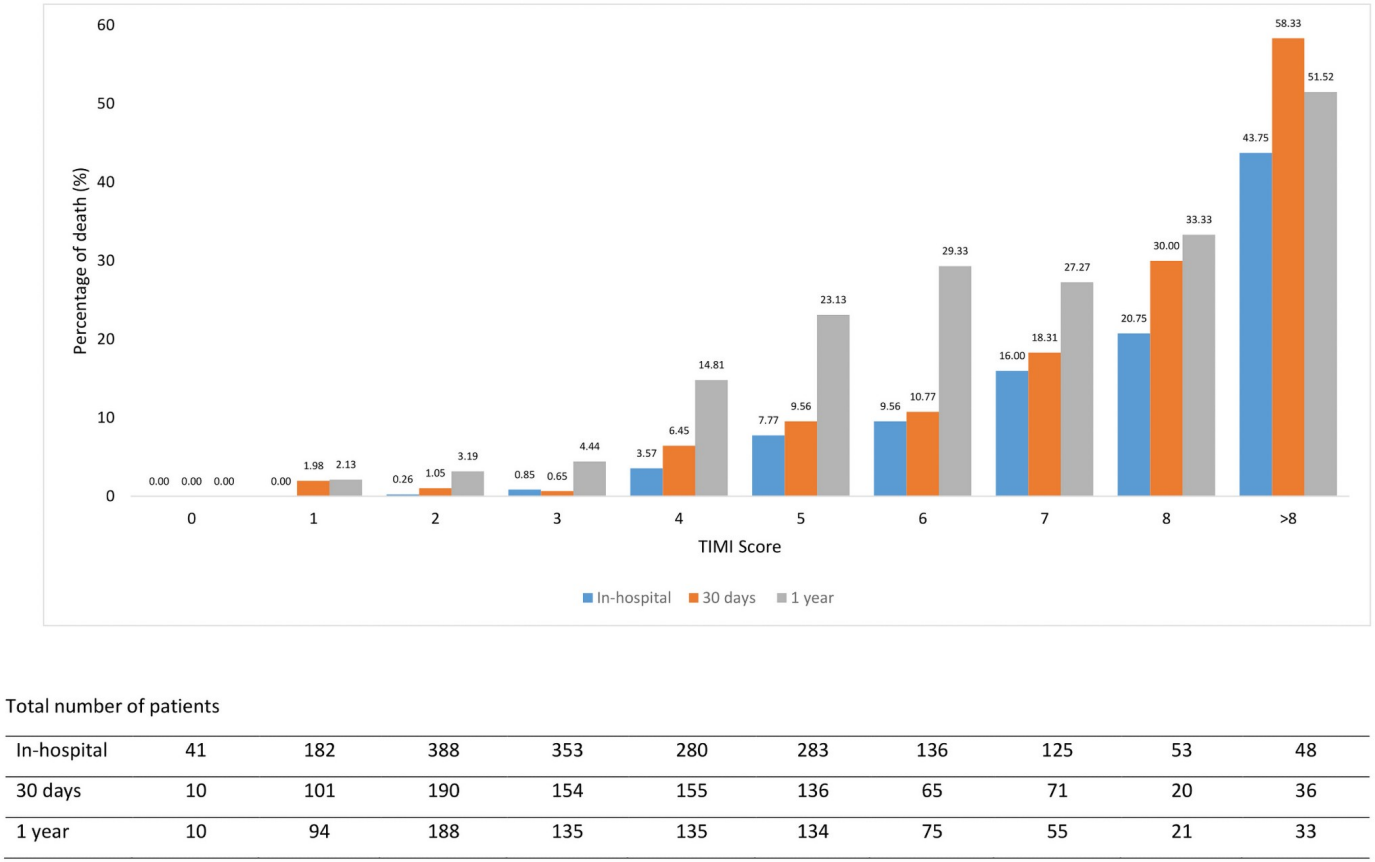
The rate of death across the risk score of TIMI.

**Fig 3:**
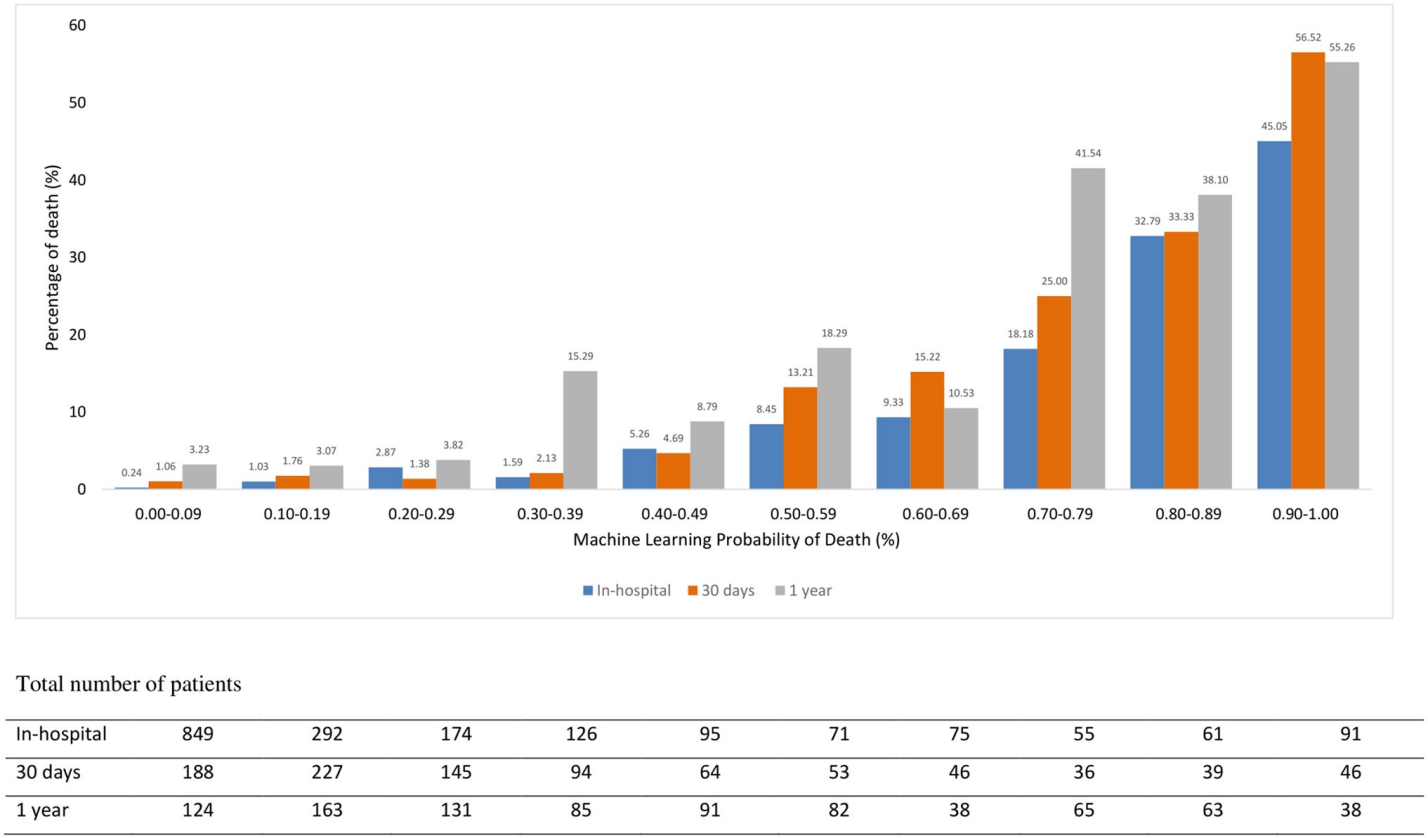
The rate of death across ML probability of death.

Figs 2 and 3 illustrate the comparison of the best ML model mortality rate against the TIMI score. ML score categorized patients as low risk with the probability <50% and high-risk stratum as ≥50%. This is equivalent to TIMI low risk of score ≤5 and a high-risk score of > 5 [5].

In the high-risk group, ML better-predicted mortality in comparison to TIMI for in-hospital death (21.94% vs 16.15%) but similar for prediction for 30 days and 1-year deaths. (25.61% vs 23.15% and 35.71% vs 34.48%).

ML predicted a better mortality rate when compared to TIMI in the lower risk group for in-hospital (1.97% vs 2.59%), 30 days (1.73% vs 3.46%) and 1-year (5.05% vs 9.35%).

Regarding the NRI for the in-hospital model, the net reclassification of patients improved using the ML produced a net reclassification improvement of 0.20 with p<0.0001 over the original TIMI risk score, that is, a 20% improved classification. NRI for 30 days reported the net reclassification of patients improved using the ML produced a net reclassification improvement of 0.19 with p<0.0001 over the original TIMI risk score, that is, a 19% improved classification. In the 1-year model, the net reclassification of patients improved using the ML produced a net reclassification improvement of 0.14 with p<0.0001 over the original TIMI risk score, that is, a 14% improved classification (Table 5).

**Table 5:** Predicted risks and reclassification of STEMI patient’s mortality between ML best model (SVMvarImp-SBE-SVM) and TIMI risk score for in-hospital, 30-days, and 1-year on 30% validation dataset.

**In-hospital**						
Individuals with events (n = 101)						
		Number of individuals		Reclassified		Net correctly reclassified (%)
		Low risk	High risk	Increased risk	Decreased risk	18.81
		Machine Learning		23	4	
	TIMI Score					
	Low risk	13	23			
	High risk	4	61			
Individuals without events (n = 1788)						
		Machine Learning		116	144	1.57
	TIMI Score					
	Low risk	1375	116			
	High risk	144	153			
Net reclassification index (NRI)						20.38
p-value						<0.0001
**30 days**						
Individuals with events (n = 75)						
		Number of individuals		Reclassified		Net correctly reclassified (%)
		Low risk	High risk	Increased risk	Decreased risk	20.00
		Machine Learning		17	2	
	TIMI Score					
	Low risk	11	17			
	High risk	2	45			
Individuals without events (n = 863)						
		Machine Learning		66	53	-1.51
	TIMI Score					
	Low risk	652	66			
	High risk	53	92			
Net reclassification index (NRI)						18.49
p-value						<0.0001
**1-year**						
Individuals with events (n = 126)						
		Number of individuals		Reclassified		Net correctly reclassified (%)
		Low risk	High risk	Increased risk	Decreased risk	23.81
		Machine Learning		44	14	
	TIMI Score					
	Low risk	21	44			
	High risk	14	47			
Individuals without events (n = 754)						
		Machine Learning		108	36	-9.55
	TIMI Score					
	Low risk	523	108			
	High risk	36	87			
Net reclassification index (NRI)						14.26
p-value						<0.0001

## Discussion

Our study is the first to show better short- and long-term mortality prediction using the ML method in a multi-ethnic Asian patient with STEMI. We demonstrated high performance on validation dataset for ML models with a combination of feature selection and classifier algorithms. Overall ML model performed better than TIMI for in-hospital, 30days and 1-year AUC of (0.88vs 0.81, 0.90 vs 0.80, 0.84 vs 0.76). SVMvarImp-SBE-SVM for in-hospital, 30 days and 1-year mortality prediction had better performance compared to RF, LR and TIMI scoring as well.

The TIMI risk score was originally developed to estimate 30 days mortality risk. In the absence of a more convenient risk score system, it has since been exploited to predict in-hospital, 30 days and 1-year mortality post-STEMI in other Asian countries as well as Mexico [2, 36–38]. This is despite its moderate accuracy for risk prediction in Asians with an AUC of 0.78 [3]. In this validation study, the Asian cohort was found to be carrying an overall higher disease burden and risk compared to the TIMI cohort. The mortality rate, however, was no different suggesting an inherent inaccuracy within the algorithm. Not only that, TIMI is known to underestimate mortality risk in the lower risk group. This may delay treatment incurring excess avoidable deaths.

TIMI risk score for STEMI consists of the following components: age; systolic blood pressure; heart rate; Killip classification; infarct location or left bundle branch block; a history of diabetes, hypertension, angina pectoris, weight, and time to reperfusion (thrombolysis or pPCI). Previous studies have modified ‘time to reperfusion’ to be ‘door-to-needle’ or ‘door-to-balloon’ time instead of ‘symptom onset to-reperfusion’ time because of inconsistencies in the reporting of symptom onset time [39]. Our study excluded some variables such as angina pectoris, weight and time to reperfusion in the model development as over 50% of data was missing. Additional parameters (Table 1) were included including ethnicity, smoking status, invasive and non-invasive treatments, lipid profile and features from the complete blood chemistry at admission.

Feature selection algorithms are essential in mortality prediction. A combination of feature selection methods with classification algorithms resulted in higher performance versus using standalone classifiers [29]. Applications of feature selection algorithms improved ML model performance using a reasonable number of predictors by reducing the predictor’s dimensionality [40]. The model performance in this study increased with the reduction in the number of predictors. Our results indicate that ML model predictive performance requires 15 predictors for in-hospital, 13 for 30 days and 12 for 1-year mortality prediction that performs better than models developed using a conventional statistical approach.

We used univariate analysis to support the relationship between variables selected from ML algorithms and outcomes (Table 1). Age, heart rate, Killip class and fasting blood glucose were ranked and selected by all short- and long-term mortality prediction ML models. Older age and higher Killip class were significant predictors of mortality [41, 42]. Age, Killip class and fasting blood glucose were also selected as a factor that affects mortality post-STEMI by ML models in previous studies [7, 19]. Glucose levels were ranked by all ML models, supporting the relationship between hyperglycemia and increased risk in mortality for patients with STEMI in the Asian population [43]. STEMI patients with higher heart rates were associated with an increased risk of mortality, even after primary PCI [44]. This may be a reflection of worse presentation (higher Killip class) or even higher pain intensity from a larger infarct.

Incorporating variables like having invasive or non-invasive management into the SVMvarImp-SBE-SVM model to predict in-hospital, 30 days and 1-year mortality yield interesting results. Invasive treatment such as PCI received by STEMI patients showed a trend towards better outcomes for in-hospital and 30 days after discharge. Mortality risk at 1-year was reduced by 40% for patients who received PCI compared to those who did not [4, 39, 45].

TIMI and GRACE scores were calculated based on data during an era where early reperfusion therapy and routine use of drug-eluting stents were not common. Non-invasive treatment predictors such as pharmacological therapy (medications including anti-hypertensive (ACE inhibitor, beta-blockers, diuretics), anti-diabetic agents (oral hypoglycaemic agents, insulin and antiplatelet) were selected for in-hospital, 30 days and 1-year mortality prediction in our study. These drugs are often prescribed in the acute setting to augment neurohumoral modulation associated with left ventricular negative remodelling. Being on these medications could signal a sicker ventricle hence the strong association with death.

Systolic and diastolic blood pressure were ranked as predictors for in-hospital, 1-year and 30 days models. Cardiogenic shock at presentation increases the risk of death. STEMI patients with cardiogenic shock who survived in-hospital death are at an increased risk of long-term death, probably as a reflection of the severity during initial admission [46].

Other CVD risk such as hypertension, diabetes, smoking and chronic renal disease, were associated with a poorer 1-year outcome. Poorly controlled CVD risk leads to an adverse systemic remodeling, leading to a plethora of cardiovascular conditions including heart failure, stroke, renal failure, and peripheral vascular disease [47].

By having continuous data collection through an electronic health records system, we were able to allow for the adaptation of ML predictive algorithm tailored to patient’s risk grouping. ML methods discussed in this study are needed to rank and select significant risk factors associated with short- and long-term STEMI mortality. Feature selection allows better interpretation of the models by restricting the scope of predictors used, selecting only those clinically relevant.

ML models in this study have demonstrated higher performance compared to TIMI scoring that was extrapolated from a Caucasian cohort. Asian patients present at a younger age with acute coronary syndromes. The average age in the GRACE registry was 61, whereas it is 58 in Malaysia and 51 in the Middle East [48]. Numerous factors are associated with differences in presentation. Hence risk scoring tools should be adapted to a specific population to better reflect the differences with greater accuracy.

Data imputation was performed to ensure the validity of the findings. We used multivariable imputation using chained equations and predictive mean matching method for data imputation instead of using machine learning-based method such as missForest in this study. The data imputation method used in this study was selected as recommended in a similar study conducted on the Swedish heart registry dataset that resulted in high model performance [19]. Moreover, Solaro et al. demonstrated that the relative performance of missForest varied with the MCAR data patterns and did not show a clear advantage. Overall, the imputation accuracy and applicability of missForest is still unclear [49]. We initially did not include patients with more than 50% missing data as it will require data imputation, which may affect our result. We do not feel it is a limitation for the population as it is still a large dataset. As the dataset had completed dataset for all follow-up time points, generation of risk calculator was possible for both ML and TIMI calculator. However, identifying factors associated with short- and long-term mortality prediction usage of complete cases would lead to more reliable findings. We went back to use an incomplete dataset and imputed data and showed similar results.

The cross-validation approach used in this study increases the efficacy of the models during model construction as it reduces the risk of model over-fitting. Also, the classification performance is highly influenced by data pre-processing and tuning of algorithms [50]. A pair-wise corrected resampled t-test was used to evaluate the differences between ML models predictive performances. The resampled t-test is a validated tool for the comparison of outcome between two classifiers [33, 34]. ML algorithms SVM and RF have demonstrated high predictive performance when combined with feature selection in mortality related studies [7, 19]. Both RF and SVM models were used to determine the list of variable importance that is an essential part of contributing to good model performance. RF, SVM and LR with SBE a feature reduction algorithm reported higher performance compared to RFE. SBE algorithm depends only on importance as an adequate term to eliminate unimportant variables one-by-one from a model [51]. Meanwhile, RFE is reported to have poor generalisation ability.

ML models in this study were validated with untouched validation data that was not used for model development, to confirm the reliability of the current study. We also demonstrated the ML model using complete sets of variables collected, without a variable selection process that resulted in a similar performance to models with feature selection. This shows that feature selection does not lead to the loss of important prognostic information.

Despite a large proportion of missing values in the original dataset, we were still able to apply both TIMI and ML algorithm and compare outcomes. This is likely because we used a hard endpoint of death that is not affected by missing values. Another possibility is that the variables extracted (15 for in-hospital, 13 for 30 days and 12 for 1-year) was sufficient to increase the model’s precision to predict death reliably.

Future study will focus on validation of the ML algorithm in real-time involving several local hospitals for continuous assessment of its reliability. Application of ML models that are population-specific together with conventional risk scoring method allows better outcome in mortality prediction, communication and increases awareness of patients that enables behavioural modifications and better management of limited resources by clinicians.

### Study limitations

This study compared the performance of an ML-based model for in-hospital, 30 days and 1-year with a clinical prognostic model that was designed for 30 days’ mortality. Its robustness would be increased had we included variables and compared them to other scoring systems such as GRACE and the Heart Score. The lack of certain variables precluded this attempt. We recognised that missing variable may result in a bias finding. We attempted to reduce this effect by applying TIMI score and ML-based score to the same population. Selection bias that exists within registries is difficult to control. We hope that future real-world study would validate our findings.

## Conclusions

In conclusion, our study illustrates the capability of ML algorithms application for feature selection and prediction of in-hospital, 30 days and 1-year population-specific mortality in STEMI patients. ML models that are population-specific demonstrate better performance compared to TIMI score. A combination of feature selection techniques with classification algorithm allows for reliable selection of significant variables and improvement in model predictive performance, with subsequent benefits by allowing effective resource allocation in the management of STEMI patients.

## Supporting information

S1 Fig The initial 50 variables ranking (a) and the selected variable ranking (b) for the in-hospital dataset. Abbreviations = ptagenotification = Age, ptrace = Race, ptsex = Gender, smokingstatus = Smoking status, chpt = History of hypertension, cdm = History of diabetes, cpremcvd = Family history of premature cardiovascular disease, cmi = History of myocardial infarction, ccap = Documented CAD, cheartfail = History of heart failure, clung = Chronic lung disease, crenal = Chronic renal disease, ccerebrovascular = Cerebrovascular disease, heartrate = Heart rate, bpsys = Systolic blood pressure, bpdias = Diastolic blood pressure, killipclass = Killip Class, tc = Total cholesterol, hdlc = High-Density Lipoprotein, ldlc = Low-Density Lipoprotein, tg = Triglycerides, fbg = Fasting blood glucose, ecgabnormstylestelev1 = ST segment elevation ≥1mm in ≥2 contiguous limb leads, ecgabnormstylestelev2 = ST segment elevation ≥2mm in ≥2 contiguous frontal leads or chest leads, ecgabnormstylestdep = ST segment depression ≥0.5mm in ≥2 contiguous leads, ecgabnormtypetwave = T-wave inversion ≥1mm, ecgabnormtypebbb = Bundle branch block, ecgabnormlocationil = Inferior leads: II, III, aVF, ecgabnormlocational = Anterior leads: V1 to V4, ecgabnormlocationll = Lateral leads: I, aVL, V5 to V6, ecgabnormlocationtp = True posterior: V1, V2, ecgabnormlocationrv = Right ventricle: ST elevation in lead V4R, fbstatus = Fibrinolytic status, pci = Percutaneous Coronary Intervention, cabg = Coronary artery bypass graft, asa = Aspirin, gpri = GP receptor inhibitor, heparin = Unfractionated heparin, lmwh = Low-molecular-weight Heparin, bb = Beta blockers, acei = ACE inhibitor, arb = Angiotensin II receptor blocker, statin = Statin, lipidla = Other lipid lowering agent, diuretic = Diuretics, calcantagonist = Calcium antagonist, oralhypogly = Oral hypoglycaemic agent, insulin = Insulin, antiarr = Anti-arrhythmic agent.
(TIF)

S2 Fig The initial 50 variables ranking (a) and the selected variable ranking (b) for the 30 days dataset. Abbreviations = ptagenotification = Age, ptrace = Race, ptsex = Gender, smokingstatus = Smoking status, chpt = History of hypertension, cdm = History of diabetes, cpremcvd = Family history of premature cardiovascular disease, cmi = History of myocardial infarction, ccap = Documented CAD, cheartfail = History of heart failure, clung = Chronic lung disease, crenal = Chronic renal disease, ccerebrovascular = Cerebrovascular disease, heartrate = Heart rate, bpsys = Systolic blood pressure, bpdias = Diastolic blood pressure, killipclass = Killip Class, tc = Total cholesterol, hdlc = High-Density Lipoprotein, ldlc = Low-Density Lipoprotein, tg = Triglycerides, fbg = Fasting blood glucose, ecgabnormstylestelev1 = ST segment elevation ≥1mm in ≥2 contiguous limb leads, ecgabnormstylestelev2 = ST segment elevation ≥2mm in ≥2 contiguous frontal leads or chest leads, ecgabnormstylestdep = ST segment depression ≥0.5mm in ≥2 contiguous leads, ecgabnormtypetwave = T-wave inversion ≥1mm, ecgabnormtypebbb = Bundle branch block, ecgabnormlocationil = Inferior leads: II, III, aVF, ecgabnormlocational = Anterior leads: V1 to V4, ecgabnormlocationll = Lateral leads: I, aVL, V5 to V6, ecgabnormlocationtp = True posterior: V1, V2, ecgabnormlocationrv = Right ventricle: ST elevation in lead V4R, fbstatus = Fibrinolytic status, pci = Percutaneous Coronary Intervention, cabg = Coronary artery bypass graft, asa = Aspirin, gpri = GP receptor inhibitor, heparin = Unfractionated heparin, lmwh = Low-molecular-weight Heparin, bb = Beta blockers, acei = ACE inhibitor, arb = Angiotensin II receptor blocker, statin = Statin, lipidla = Other lipid lowering agent, diuretic = Diuretics, calcantagonist = Calcium antagonist, oralhypogly = Oral hypoglycaemic agent, insulin = Insulin, antiarr = Anti-arrhythmic agent.
(TIF)

S3 Fig The initial 50 variables ranking (a) and the selected variable ranking (b) for the 1-year dataset. Abbreviations = ptagenotification = Age, ptrace = Race, ptsex = Gender, smokingstatus = Smoking status, chpt = History of hypertension, cdm = History of diabetes, cpremcvd = Family history of premature cardiovascular disease, cmi = History of myocardial infarction, ccap = Documented CAD, cheartfail = History of heart failure, clung = Chronic lung disease, crenal = Chronic renal disease, ccerebrovascular = Cerebrovascular disease, heartrate = Heart rate, bpsys = Systolic blood pressure, bpdias = Diastolic blood pressure, killipclass = Killip Class, tc = Total cholesterol, hdlc = High-Density Lipoprotein, ldlc = Low-Density Lipoprotein, tg = Triglycerides, fbg = Fasting blood glucose, ecgabnormstylestelev1 = ST segment elevation ≥1mm in ≥2 contiguous limb leads, ecgabnormstylestelev2 = ST segment elevation ≥2mm in ≥2 contiguous frontal leads or chest leads, ecgabnormstylestdep = ST segment depression ≥0.5mm in ≥2 contiguous leads, ecgabnormtypetwave = T-wave inversion ≥1mm, ecgabnormtypebbb = Bundle branch block, ecgabnormlocationil = Inferior leads: II, III, aVF, ecgabnormlocational = Anterior leads: V1 to V4, ecgabnormlocationll = Lateral leads: I, aVL, V5 to V6, ecgabnormlocationtp = True posterior: V1, V2, ecgabnormlocationrv = Right ventricle: ST elevation in lead V4R, fbstatus = Fibrinolytic status, pci = Percutaneous Coronary Intervention, cabg = Coronary artery bypass graft, asa = Aspirin, gpri = GP receptor inhibitor, heparin = Unfractionated heparin, lmwh = Low-molecular-weight Heparin, bb = Beta blockers, acei = ACE inhibitor, arb = Angiotensin II receptor blocker, statin = Statin, lipidla = Other lipid lowering agent, diuretic = Diuretics, calcantagonist = Calcium antagonist, oralhypogly = Oral hypoglycaemic agent, insulin = Insulin, antiarr = Anti-arrhythmic agent.
(TIF)

S1 Table The parameters setting values for optimum machine learning model performance. (DOCX)

S2 Table Patients characteristics for the in-hospital, 30-days and 1-year imputed dataset. (DOCX)
