# Supplementary figures and images for: Short- and long-term mortality prediction after an acute ST-elevation myocardial infarction (STEMI) in Asians: A machine learning approach
Source: PLoS One. 2021 Aug 2;16(8):e0254894. doi: 10.1371/journal.pone.0254894 (PMC8328310; doi:10.1371/journal.pone.0254894)

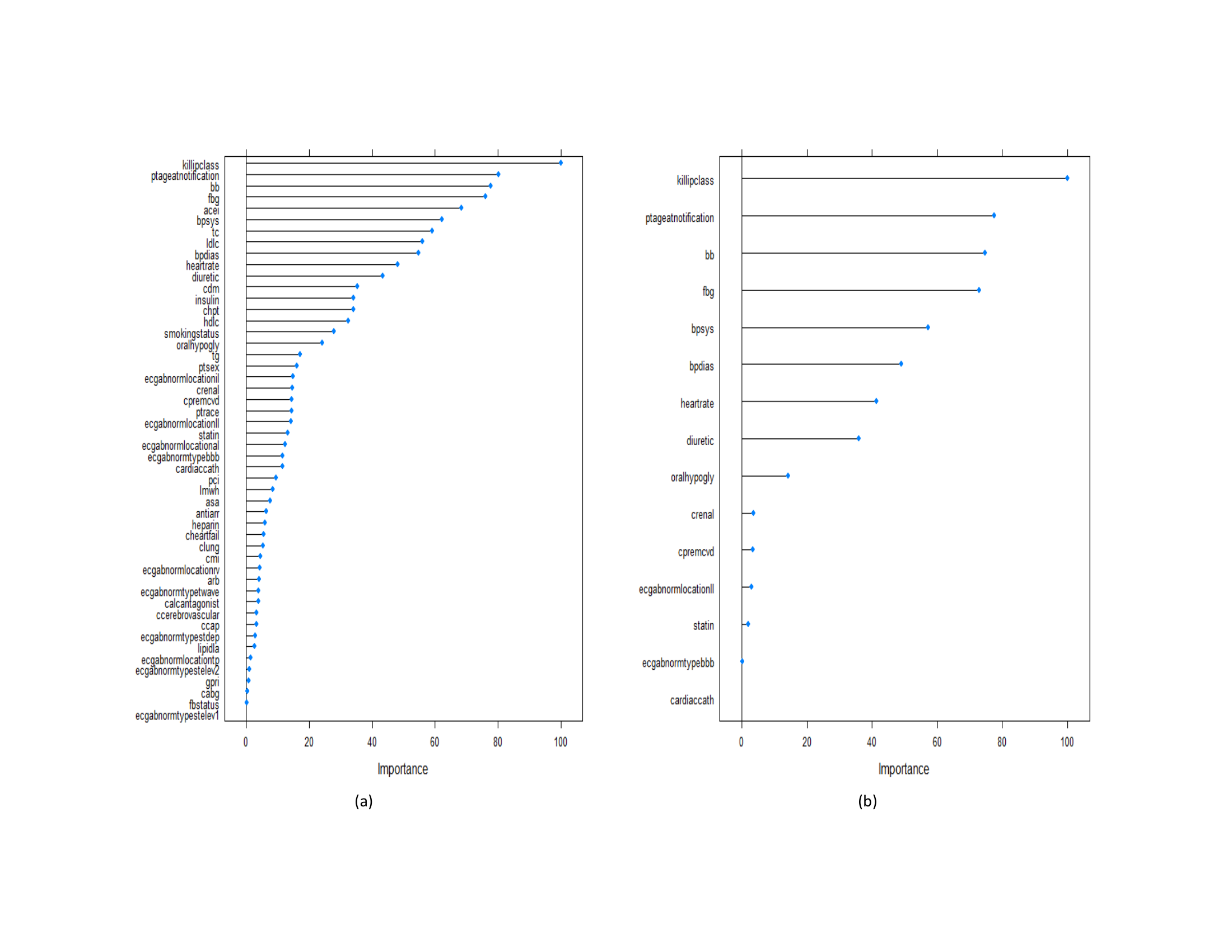

Supplement: S1 Fig — Abbreviations = ptagenotification = Age, ptrace = Race, ptsex = Gender, smokingstatus = Smoking status, chpt = History of hypertension, cdm = History of diabetes, cpremcvd = Family history of premature cardiovascular disease, cmi = History of myocardial infarction, ccap = Documented CAD, cheartfail = History of heart failure, clung = Chronic lung disease, crenal = Chronic renal disease, ccerebrovascular = Cerebrovascular disease, heartrate = Heart rate, bpsys = Systolic blood pressure, bpdias = Diastolic blood pressure, killipclass = Killip Class, tc = Total cholesterol, hdlc = High-Density Lipoprotein, ldlc = Low-Density Lipoprotein, tg = Triglycerides, fbg = Fasting blood glucose, ecgabnormstylestelev1 = ST segment elevation ≥1mm in ≥2 contiguous limb leads, ecgabnormstylestelev2 = ST segment elevation ≥2mm in ≥2 contiguous frontal leads or chest leads, ecgabnormstylestdep = ST segment depression ≥0.5mm in ≥2 contiguous leads, ecgabnormtypetwave = T-wave inversion ≥1mm, ecgabnormtypebbb = Bundle branch block, ecgabnormlocationil = Inferior leads: II, III, aVF, ecgabnormlocational = Anterior leads: V1 to V4, ecgabnormlocationll = Lateral leads: I, aVL, V5 to V6, ecgabnormlocationtp = True posterior: V1, V2, ecgabnormlocationrv = Right ventricle: ST elevation in lead V4R, fbstatus = Fibrinolytic status, pci = Percutaneous Coronary Intervention, cabg = Coronary artery bypass graft, asa = Aspirin, gpri = GP receptor inhibitor, heparin = Unfractionated heparin, lmwh = Low-molecular-weight Heparin, bb = Beta blockers, acei = ACE inhibitor, arb = Angiotensin II receptor blocker, statin = Statin, lipidla = Other lipid lowering agent, diuretic = Diuretics, calcantagonist = Calcium antagonist, oralhypogly = Oral hypoglycaemic agent, insulin = Insulin, antiarr = Anti-arrhythmic agent. (TIF) [file pone.0254894.s001.tif]

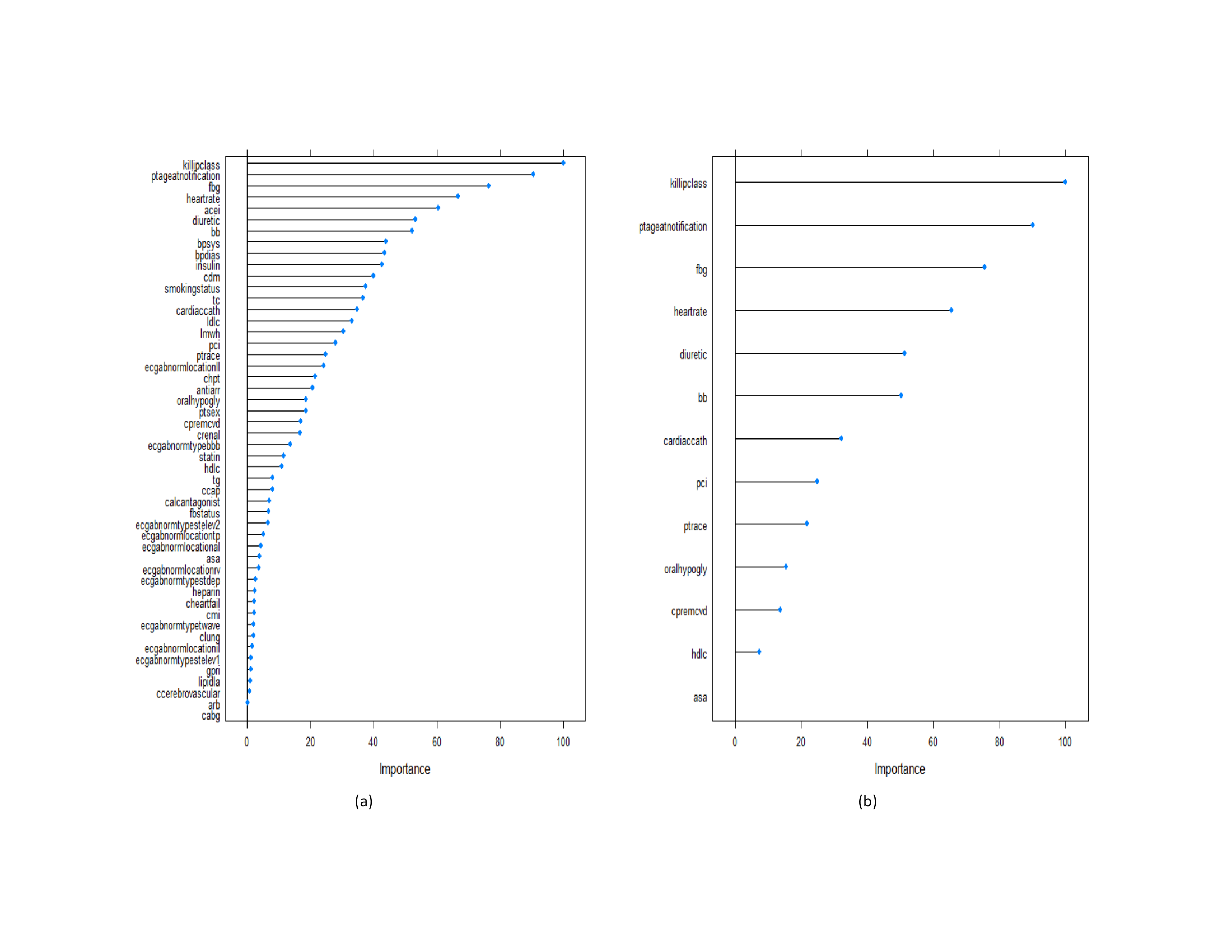

Supplement: S2 Fig — Abbreviations = ptagenotification = Age, ptrace = Race, ptsex = Gender, smokingstatus = Smoking status, chpt = History of hypertension, cdm = History of diabetes, cpremcvd = Family history of premature cardiovascular disease, cmi = History of myocardial infarction, ccap = Documented CAD, cheartfail = History of heart failure, clung = Chronic lung disease, crenal = Chronic renal disease, ccerebrovascular = Cerebrovascular disease, heartrate = Heart rate, bpsys = Systolic blood pressure, bpdias = Diastolic blood pressure, killipclass = Killip Class, tc = Total cholesterol, hdlc = High-Density Lipoprotein, ldlc = Low-Density Lipoprotein, tg = Triglycerides, fbg = Fasting blood glucose, ecgabnormstylestelev1 = ST segment elevation ≥1mm in ≥2 contiguous limb leads, ecgabnormstylestelev2 = ST segment elevation ≥2mm in ≥2 contiguous frontal leads or chest leads, ecgabnormstylestdep = ST segment depression ≥0.5mm in ≥2 contiguous leads, ecgabnormtypetwave = T-wave inversion ≥1mm, ecgabnormtypebbb = Bundle branch block, ecgabnormlocationil = Inferior leads: II, III, aVF, ecgabnormlocational = Anterior leads: V1 to V4, ecgabnormlocationll = Lateral leads: I, aVL, V5 to V6, ecgabnormlocationtp = True posterior: V1, V2, ecgabnormlocationrv = Right ventricle: ST elevation in lead V4R, fbstatus = Fibrinolytic status, pci = Percutaneous Coronary Intervention, cabg = Coronary artery bypass graft, asa = Aspirin, gpri = GP receptor inhibitor, heparin = Unfractionated heparin, lmwh = Low-molecular-weight Heparin, bb = Beta blockers, acei = ACE inhibitor, arb = Angiotensin II receptor blocker, statin = Statin, lipidla = Other lipid lowering agent, diuretic = Diuretics, calcantagonist = Calcium antagonist, oralhypogly = Oral hypoglycaemic agent, insulin = Insulin, antiarr = Anti-arrhythmic agent. (TIF) [file pone.0254894.s002.tif]

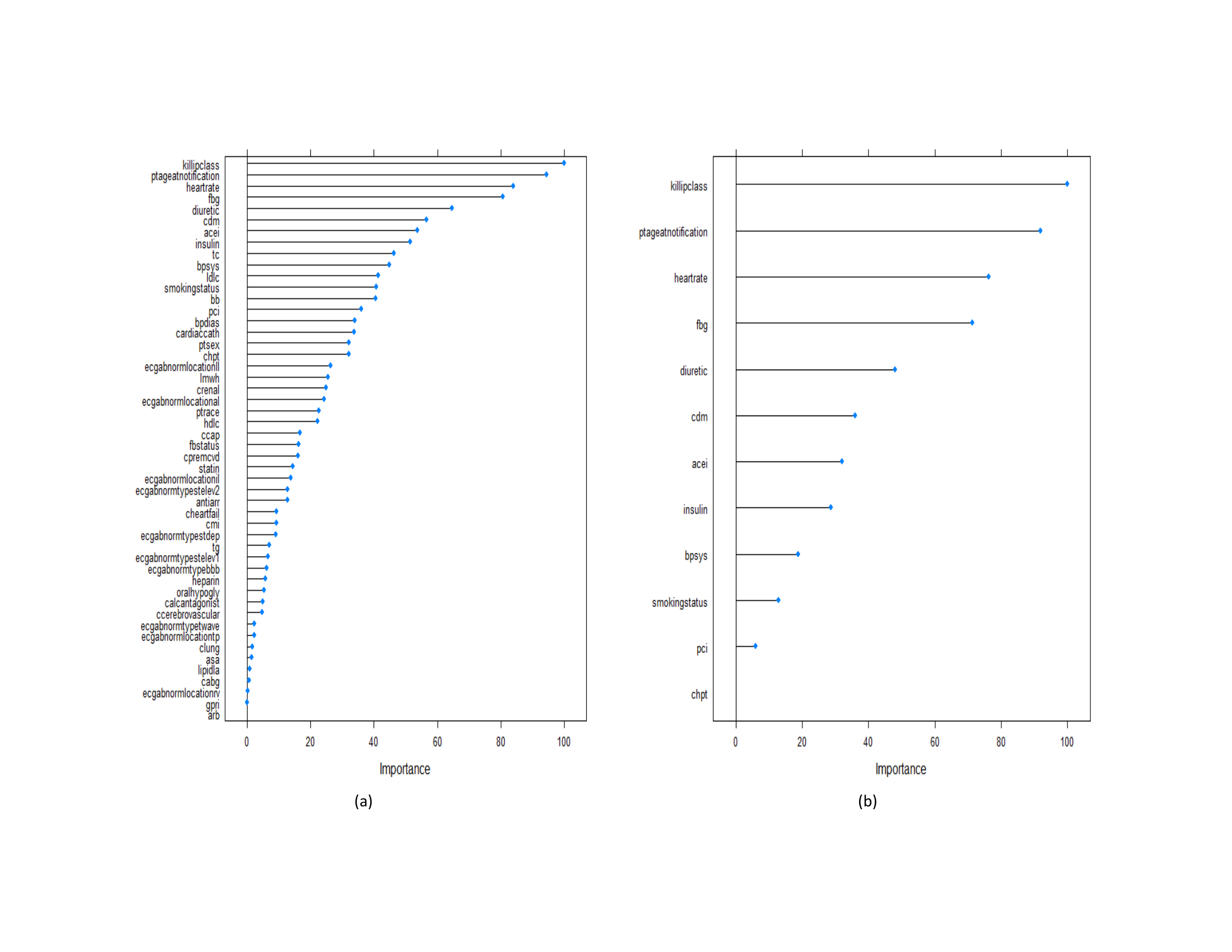

Supplement: S3 Fig — Abbreviations = ptagenotification = Age, ptrace = Race, ptsex = Gender, smokingstatus = Smoking status, chpt = History of hypertension, cdm = History of diabetes, cpremcvd = Family history of premature cardiovascular disease, cmi = History of myocardial infarction, ccap = Documented CAD, cheartfail = History of heart failure, clung = Chronic lung disease, crenal = Chronic renal disease, ccerebrovascular = Cerebrovascular disease, heartrate = Heart rate, bpsys = Systolic blood pressure, bpdias = Diastolic blood pressure, killipclass = Killip Class, tc = Total cholesterol, hdlc = High-Density Lipoprotein, ldlc = Low-Density Lipoprotein, tg = Triglycerides, fbg = Fasting blood glucose, ecgabnormstylestelev1 = ST segment elevation ≥1mm in ≥2 contiguous limb leads, ecgabnormstylestelev2 = ST segment elevation ≥2mm in ≥2 contiguous frontal leads or chest leads, ecgabnormstylestdep = ST segment depression ≥0.5mm in ≥2 contiguous leads, ecgabnormtypetwave = T-wave inversion ≥1mm, ecgabnormtypebbb = Bundle branch block, ecgabnormlocationil = Inferior leads: II, III, aVF, ecgabnormlocational = Anterior leads: V1 to V4, ecgabnormlocationll = Lateral leads: I, aVL, V5 to V6, ecgabnormlocationtp = True posterior: V1, V2, ecgabnormlocationrv = Right ventricle: ST elevation in lead V4R, fbstatus = Fibrinolytic status, pci = Percutaneous Coronary Intervention, cabg = Coronary artery bypass graft, asa = Aspirin, gpri = GP receptor inhibitor, heparin = Unfractionated heparin, lmwh = Low-molecular-weight Heparin, bb = Beta blockers, acei = ACE inhibitor, arb = Angiotensin II receptor blocker, statin = Statin, lipidla = Other lipid lowering agent, diuretic = Diuretics, calcantagonist = Calcium antagonist, oralhypogly = Oral hypoglycaemic agent, insulin = Insulin, antiarr = Anti-arrhythmic agent. (TIF) [file pone.0254894.s003.tif]
