# Supplementary material for: Short- and long-term mortality prediction after an acute ST-elevation myocardial infarction (STEMI) in Asians: A machine learning approach
Source: PLoS One. 2021 Aug 2;16(8):e0254894. doi: 10.1371/journal.pone.0254894 (PMC8328310; doi:10.1371/journal.pone.0254894)
Supplement: S1 Table — (DOCX) [file pone.0254894.s004.docx]

**S1 Table: The parameters setting values for optimum the machine learning model performance.**

| **Random Forest** |
| --- |
| Seed = 42 |
| mtry =$\surd n$ |
| Number of tree = 1000 |
| Pre-processing = centre and scale |
| Cross-validation=10 |
| Number of iteration = 3 |
|  |
| **Support Vector Machine** |
| Seed = 42 |
| Pre-processing = centre and scale |
| C-value = 0.25 |
| Sigma value = 0.01 |
| Cross-validation=10 |
|  |
| **Logistic Regression** |
| Seed = 42 |
| Method= glm |
| Cross-validation=10 |
| Tune length = 10 |
| Family = binomial |
