# Supplementary material for: Short- and long-term mortality prediction after an acute ST-elevation myocardial infarction (STEMI) in Asians: A machine learning approach
Source: PLoS One. 2021 Aug 2;16(8):e0254894. doi: 10.1371/journal.pone.0254894 (PMC8328310; doi:10.1371/journal.pone.0254894)
Supplement: S2 Table — (DOCX) [file pone.0254894.s005.docx]

**S2 Table: Patients characteristics for the in-hospital, 30-days and 1-year imputed dataset.**

| Variables | Description | In-hospital | | | | 30 days | | | | 1 year | | | |
| --- | --- | --- | --- | --- | --- | --- | --- | --- | --- | --- | --- | --- | --- |
|  |  | Total | Survival | Non-survival | p-value | Total | Survival | Non-survival | p-value | Total | Survival | Non-survival | p-value |
| N |  | 17296 | 16140 (93.3) | 1156 (6.7) |  | 8261 | 7947 (96.2) | 314 (3.8) |  | 7821 | 7374 (94.2) | 447 (5.8) |  |
| Age |  | 56.40±11.77 | 55.89±11.60 | 63.59±11.89 | **<0.0001** | 56.74±11.80 | 56.46±11.69 | 63.80±12.22 | **<0.0001** | 56.63±11.79 | 56.26±11.68 | 62.80±11.88 | **<0.0001** |
| Race | Malay | 10035 (58.0) | 9318 (57.7) | 717 (62.0) | **<0.0001** | 4787 (57.9) | 4597 (57.8) | 190 (60.5) | 0.001 | 4503 (57.6) | 4231 (57.4) | 272 (60.9) | **<0.0001** |
|  | Chinese | 3130 (18.1) | 2915 (18.1) | 215 (18.6) |  | 1412 (17.1) | 1345 (16.9) | 67 (21.3) |  | 1340 (17.1) | 1258 (17.1) | 82 (18.3) |  |
|  | Indian | 2888 (16.7) | 2713 (16.8) | 175 (15.1) |  | 1365 (16.5) | 1317 (16.6) | 48 (15.3) |  | 1296 (16.6) | 1213 (16.4) | 83 (18.6) |  |
|  | Others | 1243 (7.2) | 1194 (7.4) | 49 (4.2) |  | 697 (8.4) | 688 (8.7) | 9 (2.9) |  | 682 (8.7) | 672 (9.1) | 10 (2.2) |  |
| Gender | Male | 14851 (85.9) | 13985 (86.6) | 866 (74.9) | **<0.0001** | 7110 (86.1) | 6866 (86.4) | 244 (77.7) | **<0.0001** | 6760 (86.4) | 6422 (87.1) | 338 (75.6) | **<0.0001** |
|  | Female | 2445 (14.1) | 2155 (13.4) | 290 (25.1) |  | 1151 (13.9) | 1081 (13.6) | 70 (22.3) |  | 1061 (13.6) | 952 (12.9) | 109 (24.4) |  |
| Smoking status | Never | 5356 (31.0) | 4864 (30.1) | 492 (42.6) | **<0.0001** | 2608 (31.6) | 2469 (31.10 | 139 (44.3) | **<0.0001** | 2432 (31.1) | 2232 (30.3) | 200 (44.7) | **<0.0001** |
|  | Former (quit tobacco > 30days) | 2985 (17.3) | 2752 (17.1) | 233 (20.2) |  | 1381 (16.7) | 1318 (16.6) | 63 (20.1) |  | 1297 (16.6) | 1219 (16.5) | 78 (17.4) |  |
|  | Current (tobacco < 30days) | 8955 (51.8) | 8524 (52.8) | 431 (37.3) |  | 4272 (51.7) | 4160 (52.3) | 112 (15.7) |  | 4092 (52.3) | 3923 (53.2) | 169 (37.8) |  |
| History of hypertension |  | 9662 (55.9) | 8851 (54.8) | 811 (70.2) | **<0.0001** | 4614 (55.9) | 4408 (55.5) | 206 (65.6) | **<0.0001** | 4377 (56.0) | 4086 (55.4) | 291 (65.1) | **<0.0001** |
| History of diabetes |  | 6952 (40.2) | 6318 (39.1) | 634 (54.8) | **<0.0001** | 3417 (41.4) | 3237 (40.7) | 180 (57.3) | **<0.0001** | 3185 (40.7) | 2927 (39.7) | 258 (57.7) | **<0.0001** |
| Family history of premature cardiovascular disease |  | 2432 (14.1) | 2320 (14.4) | 112 (9.7) | **<0.0001** | 1165 (14.1) | 1137 (14.3) | 28 (8.9) | 0.007 | 1079 (13.8) | 1039 (14.1) | 40 (8.9) | 0.002 |
| History of myocardial infarction |  | 2142 (12.4) | 1989 (12.3) | 153 (13.2) | 0.363 | 1052 (12.7) | 1012 (12.7) | 40 (12.7) | 0.998 | 968 (12.4) | 916 (12.4) | 52 (11.6) | 0.623 |
| Documented CAD |  | 1799 (10.4) | 1666 (10.3) | 133 (11.5) | 0.203 | 1018 (12.3) | 973 (12.2) | 45 (14.3) | 0.270 | 942 (12.0) | 869 (11.8) | 73 (16.3) | 0.004 |
| History of heart failure |  | 496 (2.9) | 400 (2.5) | 96 (8.3) | **<0.0001** | 211 (2.6) | 198 (2.5) | 13 (4.1) | 0.069 | 183 (2.3) | 165 (2.2) | 18 (4.0) | 0.015 |
| Chronic lung disease |  | 385 (2.2) | 334 (2.1) | 51 (4.4) | **<0.0001** | 186 (2.3) | 175 (2.2) | 11 (3.5) | 0.127 | 164 (2.1) | 146 (2.0) | 18 (4.0) | 0.003 |
| Chronic renal disease |  | 673 (3.9) | 541 (3.4) | 132 (11.4) | **<0.0001** | 347 (4.2) | 308 (3.9) | 39 (12.4) | **<0.0001** | 309 (4.0) | 258 (3.5) | 51 (11.4) | **<0.0001** |
| Cerebrovascular disease |  | 576 (3.3) | 522 (3.2) | 54 (4.7) | 0.009 | 275 (3.3) | 262 (3.3) | 13 (4.1) | 0.414 | 258 (3.3) | 236 (3.2) | 22 (4.9) | 0.048 |
| Heart rate |  | 83.18±21.71 | 82.49±21.12 | 92.80±26.90 | **<0.0001** | 83.38±21.68 | 82.92±21.32 | 94.91±26.81 | **<0.0001** | 83.04±21.43 | 82.52±21.11 | 91.69±24.62 | **<0.0001** |
| Systolic blood pressure |  | 133.56±28.93 | 134.59±28.46 | 119.27±31.67 | **<0.0001** | 133.59±29.06 | 133.84±28.94 | 127.08±31.30 | **<0.0001** | 133.77±28.78 | 133.98±28.64 | 130.30±30.97 | 0.009 |
| Diastolic blood pressure |  | 81.20±58.90 | 81.78±60.57 | 73.04±24.81 | **<0.0001** | 80.58±18.85 | 80.73±18.78 | 16.67±20.24 | **<0.0001** | 80.77±18.72 | 8094±18.64 | 78.12±19.72 | 0.002 |
| Killip class | I | 11091 (64.1) | 10798 (66.9) | 293 (25.3) | **<0.0001** | 5160 (62.5) | 5069 (63.8) | 91 (29.0) | **<0.0001** | 4960 (63.4) | 4781 (64.8) | 179 (40.0) | **<0.0001** |
|  | II | 3317 (19.2) | 3092 (19.2) | 225 (19.5) |  | 1496 (18.1) | 1434 (18.0) | 62 (19.7) |  | 1395 (17.8) | 1286 (17.4) | 109 (24.4) |  |
|  | III | 750 (4.3) | 620 (3.8) | 130 (11.2) |  | 378 (4.6) | 335 (4.2) | 43 (13.7) |  | 336 (4.3) | 295 (4.0) | 41 (9.2) |  |
|  | IV | 2138 (12.4) | 1630 (10.1) | 508 (43.9) |  | 1227 (14.9) | 1109 (14.0) | 118 (37.6) |  | 1130 (14.4) | 1012 (13.7) | 118 (26.4) |  |
| Total cholesterol |  | 5.67±13.60 | 5.72±14.06 | 4.98±2.58 | 0.073 | 5.24±1.44 | 5.25±1.43 | 4.94±1.66 | **<0.0001** | 5.28±1.44 | 5.30±1.44 | 4.97±1.56 | **<0.0001** |
| HDL |  | 1.16±2.70 | 1.16±2.68 | 1.15±2.94 | 0.839 | 1.09±0.34 | 1.09±0.34 | 1.08±0.35 | 0.575 | 1.09±0.34 | 1.09±0.34 | 1.07±0.35 | 0.274 |
| LDL |  | 3.80±12.06 | 3.76±9.53 | 4.39±30.16 | 0.087 | 3.45±3.48 | 3.46±3.53 | 3.16±1.46 | 0.126 | 3.43±1.28 | 3.44±1.27 | 3.16±1.37 | **<0.0001** |
| Triglycerides |  | 1.91±6.43 | 1.87±4.98 | 2.43±16.48 | 0.004 | 1.69±0.99 | 1.69±1.00 | 1.63±0.86 | 0.292 | 1.71±1.04 | 1.72±1.06 | 1.62±0.81 | 0.057 |
| Fasting blood glucose |  | 8.71±4.77 | 8.48±4.54 | 11.83±6.55 | **<0.0001** | 8.77±4.40 | 8.65±4.26 | 11.64±6.48 | **<0.0001** | 8.70±4.32 | 8.58±4.19 | 10.74±5.78 | **<0.0001** |
| ECG abnormalities type | ST segment elevation ≥1mm in ≥2 contiguous limb leads | 8158 (47.2) | 7634 (47.3) | 524 (45.3) | 0.195 | 4037 (48.9) | 3893 (49.0) | 144 (45.9) | 0.277 | 3848 (49.2) | 3638 (49.3) | 210 (47.0) | 0.333 |
|  | ST segment elevation ≥2mm in ≥2 contiguous frontal leads or chest leads | 9758 (56.4) | 9079 (56.3) | 679 (58.7) | 0.100 | 4548 (55.1) | 4353 (54.8) | 195 (62.1) | 0.010 | 4280 (54.7) | 4005 (54.3) | 275 (61.5) | 0.003 |
|  | ST segment depression ≥0.5mm in ≥2 contiguous leads | 1812 (10.5) | 1664 (10.3) | 148 (12.8) | 0.008 | 779 (9.4) | 745 (9.4) | 34 (10.8) | 0.387 | 731 (9.3) | 681 (9.2) | 50 (11.2) | 0.169 |
|  | T-wave inversion ≥1mm | 1004 (5.8) | 952 (5.9) | 52 (4.5) | 0.049 | 459 (5.6) | 437 (5.5) | 22 (7.0) | 0.253 | 434 (5.5) | 395 (5.4) | 39 (8.7) | 0.003 |
|  | Bundle branch block | 388 (2.2) | 306 (1.9) | 82 (7.1) | **<0.0001** | 175 (2.1) | 154 (1.9) | 21 (6.7) | **<0.0001** | 148 (1.9) | 126 (1.7) | 22 (4.9) | **<0.0001** |
| ECG abnormalities location | Inferior leads: II, III, aVF | 8189 (47.3) | 7694 (47.7) | 495 (42.8) | 0.001 | 3956 (47.9) | 3833 (48.2) | 123 (39.2) | 0.002 | 3777 (48.3) | 3583 (48.6) | 194 (43.4) | 0.033 |
|  | Anterior leads: V1 to V4 | 9248 (53.7) | 8606 (53.3) | 678 (58.7) | **<0.0001** | 4351 (52.7) | 4150 (52.2) | 201 (64.0) | **<0.0001** | 4081 (52.2) | 3809 (51.7) | 272 (60.9) | **<0.0001** |
|  | Lateral leads : I, aVL, V5 to V6 | 3683 (21.3) | 3370 (20.9) | 313 (27.1) | **<0.0001** | 1829 (22.1) | 1724 (21.7) | 105 (33.4) | **<0.0001** | 1737 (22.2) | 1593 (21.6) | 144 (32.2) | **<0.0001** |
|  | True posterior: V1, V2 | 1337 (7.7) | 1241 (7.7) | 96 (8.3) | 0.449 | 603 (7.3) | 578 (7.3) | 25 (8.0) | 0.645 | 581 (7.4) | 549 (7.4) | 32 (7.2) | 0.823 |
|  | Right ventricle: ST elevation in lead V4R | 1229 (7.1) | 1142 (7.1) | 87 (7.5) | 0.565 | 595 (7.2) | 568 (7.1) | 27 (8.6) | 0.329 | 561 (7.2) | 522 (7.1) | 39 (8.7) | 0.190 |
| Fb status |  | 12472 (72.1) | 11670 (72.3) | 802 (69.4) | 0.032 | 4259 (51.6) | 4063 (51.1) | 196 (62.4) | **<0.0001** | 5341 (68.3) | 5053 (68.5) | 288 (64.4) | 0.071 |
| Cardiac catheterization |  | 7440 (43.0) | 7078 (43.9) | 362 (31.3) | **<0.0001** | 4248 (51.4) | 4115 (51.8) | 133 (42.4) | 0.001 | 4026 (51.5) | 3836 (52.0) | 190 (42.5) | **<0.0001** |
| PCI |  | 6176 (35.7) | 5885 (36.5) | 291 (25.2) | **<0.0001** | 3495 (42.3) | 3384 (42.6) | 111 (35.4) | 0.011 | 3333 (42.6) | 3187 (43.2) | 146 (32.7) | **<0.0001** |
| CABG |  | 123 (0.7) | 117 (0.7) | 6 (0.5) | 0.421 | 106 (1.3) | 99 (1.2) | 7 (2.2) | 0.129 | 76 (1.0) | 72 (1.0) | 4 (0.9) | 0.864 |
| ASA |  | 16829 (97.3) | 15793 (97.9) | 1036 (89.6) | **<0.0001** | 8059 (97.6) | 7757 (97.6) | 302 (96.2) | 0.107 | 7634 (97.6) | 7200 (97.6) | 434 (97.1) | 0.461 |
| GP receptor inhibitor |  | 593 (3.4) | 541 (3.4) | 52 (4.5) | 0.039 | 201 (2.4) | 194 (2.4) | 7 (2.2) | 0.811 | 205 (2.6) | 195 (2.6) | 10 (2.2) | 0.601 |
| Heparin |  | 2794 (16.2) | 2583 (16.0) | 211 (18.3) | 0.045 | 1552 (18.8) | 1493 (18.8) | 59 (18.8) | 0.999 | 1503 (19.2) | 1433 (19.4) | 70 (15.7) | 0.049 |
| LMWH |  | 4929 (28.5) | 4545 (28.2) | 384 (33.2) | **<0.0001** | 1532 (18.5) | 1441 (18.1) | 91 (29.0) | **<0.0001** | 1295 (16.6) | 1195 (16.2) | 100 (22.4) | 0.001 |
| Beta blockers |  | 10914 (63.1) | 10609 (65.7) | 305 (26.4) | **<0.0001** | 5009 (60.6) | 4873 (61.3) | 136 (43.3) | **<0.0001** | 4704 (60.1) | 4490 (60.9) | 214 (47.9) | **<0.0001** |
| ACE inhibitor |  | 9093 (52.6) | 8832 (54.7) | 261 (22.6) | **<0.0001** | 4016 (48.6) | 3928 (49.4) | 88 (28.0) | **<0.0001** | 3765 (48.1) | 3632 (49.3) | 133 (29.8) | **<0.0001** |
| Angiotensin II receptor blocker |  | 613 (3.5) | 584 (3.6) | 29 (2.5) | 0.049 | 209 (2.5) | 202 (2.5) | 7 (2.2) | 0.729 | 181 (2.3) | 172 (2.3) | 9 (2.0) | 0.663 |
| Statin |  | 16078 (93.0) | 15169 (94.0) | 909 (78.6) | **<0.0001** | 7688 (93.1) | 7399 (93.1) | 289 (92.0) | 0.466 | 7289 (93.2) | 6880 (93.3) | 409 (91.5) | 0.142 |
| Other lipid lowering agent |  | 475 (2.7) | 452 (2.8) | 23 (2.0) | 0.103 | 168 (2.0) | 165 (2.1) | 3 (1.0) | 0.168 | 147 (1.9) | 137 (1.9) | 10 (2.2) | 0.566 |
| Diuretics |  | 4064 (23.5) | 3647 (22.6) | 417 (36.1) | **<0.0001** | 2099 (25.4) | 1950 (24.5) | 149 (47.5) | **<0.0001** | 1876 (24.0) | 1686 (22.9) | 190 (42.5) | **<0.0001** |
| Calcium antagonist |  | 1062 (6.1) | 1003 (6.2) | 59 (5.1) | 0.129 | 488 (5.9) | 474 (6.0) | 14 (4.5) | 0.267 | 418 (5.3) | 390 (5.3) | 28 (6.3) | 0.373 |
| Oral hypoglycaemic agent |  | 3418 (19.8) | 3297 (20.4) | 121 (10.5) | **<0.0001** | 1406 (17.0) | 1365 (17.2) | 41 (13.1) | 0.057 | 1305 (16.7) | 1227 (16.6) | 78 (17.4) | 0.656 |
| Insulin |  | 4498 (26.0) | 4098 (25.3) | 409 (35.4) | **<0.0001** | 2251 (27.2) | 2109 (26.5) | 142 (45.2) | **<0.0001** | 2087 (26.7) | 1895 (25.7) | 192 (43.0) | **<0.0001** |
| Anti-arrhythmic agent |  | 1231 (7.10 | 1049 (6.5) | 182 (15.7) | **<0.0001** | 592 (7.20 | 544 (6.8) | 48 (15.3) | **<0.0001** | 505 (6.5) | 464 (6.3) | 41 (9.2) | 0.016 |

Abbreviations: CAD = coronary artery disease, HDL = high-density lipoprotein, LDL = low-density lipoprotein, ECG = electrocardiogram, FB = fibrinolytic therapy, PCI = percutaneous coronary intervention, CABG = coronary artery bypass graft, ASA = acetylsalicylic acid (aspirin), GP = glycoprotein, LMWH = low-molecular-weight heparin, ACE = Angiotensin-converting enzyme.

Data are shown as n (%) for categorical variables and mean ± SD for continuous variables.

p value is statistically highly significant as p < 0.001.
